# Circadian PERformance in breast cancer: a germline and somatic genetic study of *PER3*^VNTR^ polymorphisms and gene co-expression

**DOI:** 10.1038/s41523-021-00329-2

**Published:** 2021-09-10

**Authors:** Jaume Fores-Martos, Raimundo Cervera-Vidal, Julia Sierra-Roca, Carlos Lozano-Asencio, Vita Fedele, Sten Cornelissen, Hege Edvarsen, Irene Tadeo-Cervera, Pilar Eroles, Ana Lluch, Rafa Tabares-Seisdedos, Antonio Falcó, Laura J. Van’t Veer, Marjanka Schmidt, David A. Quigley, Anne-Lise Børresen-Dale, Vessela N. Kristensen, Allan Balmain, Joan Climent

**Affiliations:** 1 ESI International Chair at CEU-UCH, CEU Universities, Valencia, Spain; 2 Biomedical Research Networking Center of Mental Health (CIBERSAM), Madrid, Spain; 3 INCLIVA Research Institute. Hospital Clínico Universitario de Valencia, Valencia, Spain; 4 Servicio de Pediatria, Hospital La Fe, Valencia, Spain; 5 Digestive Molecular Clinical Oncology Research Unit, Section of Medical Oncology, Department of Medicine, University of Verona, Verona, Italy; 6 Division of Molecular Pathology, The Netherlands Cancer Institute - Antoni van Leeuwenhoek Hospital, Amsterdam, The Netherlands; 7 Department of Genetics, Institute for Cancer Research, Oslo University Hospital Radiumhospitalet, Oslo, Norway; 8 Departamento de Producción y Sanidad Animal, Salud Pública Veterinaria y Ciencia y Tecnología de los Alimentos. Facultad de Veterinaria, Universidad CEU Cardenal Herrera. CEU Universities, Valencia, Spain; 9 Department of Medicine, Faculty of Medicine, University of Valencia, Valencia, Spain; 10 Departamento de Matemáticas, Física y Ciencias Tecnológicas, Escuela Superior de Enseñanzas Técnicas, Universidad CEU Cardenal Herrera, CEU Universities, Valencia, Spain; 11 UCSF Helen Diller Family Comprehensive Cancer Center, University of California San Francisco, San Francisco, CA USA; 12 Departments of Urology and Epidemiology & Biostatistics, University of California San Francisco, Helen Diller Family Comprehensive Cancer Center, San Francisco, CA USA

**Keywords:** Genetic association study, Cancer genetics

## Abstract

Polymorphisms in the *PER3* gene have been associated with several human disease phenotypes, including sleep disorders and cancer. In particular, the long allele of a variable number of tandem repeat (VNTR) polymorphism has been previously linked to an increased risk of breast cancer. Here we carried out a combined germline and somatic genetic analysis of the role of the *PER3*^VNRT^ polymorphism in breast cancer. The combined data from 8284 individuals showed a non-significant trend towards increased breast cancer risk in the 5-repeat allele homozygous carriers (OR = 1.17, 95% CI: 0.97–1.42). We observed allelic imbalance at the *PER3* locus in matched blood and tumor DNA samples, showing a significant retention of the long variant (risk) allele in tumor samples, and a preferential loss of the short repetition allele (*p* = 0.0005). Gene co-expression analysis in healthy and tumoral breast tissue samples uncovered significant associations between *PER3* expression levels with those from genes which belong to several cancer-associated pathways. Finally, relapse-free survival (RFS) analysis showed that low expression levels of *PER3* were linked to a significant lower RSF in luminal A (*p* = 3 × 10^−12^) but not in the rest of breast cancer subtypes.

## Introduction

The circadian clock is a cell-autonomous mechanism implicated in the control of numerous physiological processes^1–3^, which, in humans, it is adjusted to the earth daily cycle by the light captured at the retina^3^. *PER3* is part of the negative branch of the primary molecular circadian system feedback loop^3–6^ whose expression oscillates in peripheral tissues and organs^7–10^. *PER3* is located on 1p36 chromosomal region, a commonly deleted region in human cancer, and especially in breast tumors^11–13^.

We have previously shown that *PER3* deletion is associated with tumor recurrence in patients with estrogen receptor (ER) positive breast cancers treated with tamoxifen, in addition, we also observed that low expression levels of *PER3* may serve as a predictor of the probability of breast tumor recurrence in patients with ER-positive tumors^11^.

To look for potential *PER3* inactivating mutations in breast cancer, we formerly sequenced the complete coding region of *PER3* in human breast cancer cell lines, and although no clear pathogenic mutations were identified, one of the polymorphic variants of *PER3*, a variable number of tandem repeats (*PER3*^VNTR^) was observed^11^.

The primate specific *PER3*^VNTR^ polymorphism consists in a 54-nucleotide coding-region located at exon 18 that is repeated 4 or 5 times in humans. The *PER3*^VNTR^ polymorphism has been linked to an increased risk of colorectal adenoma formation and breast cancer^14,15^.

Zhu et al. reported that the *PER3* 5-repetition allele was associated to an increased risk of breast cancer among premenopausal Caucasian women in a case–control study including 389 cases and 432 controls^15^. This finding was however not replicated either by Dai et al. in a larger Chinese population case–control study including 1519 cases and 1600 controls^16^, nor by Wirth et al. in a small Indian population study^17^.

To determine if *PER3* 5-repeat allele is associated with an increased breast cancer risk we carried out a case–control study using two independent cohorts derived from Norway (Oslo University Hospital) and Netherlands (Netherlands Cancer Institute), and combined our results through meta-analysis with previously published data. Overall, we obtained *PER3* genotypes for, 5931 samples, including, 2420 cases, 2207 controls, and 1304 breast tumor samples from which 329 presented matched blood genotypes derived from a subset of patients included in the case group. *PER3*^VNRT^ genotypes were also obtained for a collection of 52 breast cancer cell lines. For the subset of samples for which we had patient matched blood and tumor data, we determine if changes in the *PER3*^VNTR^ genotype between germline and tumor samples were taking place.

Finally, given the reported role of *PER3* low expression levels in breast cancer tumor recurrence^11^, we aimed to characterize *PER3* placement in the context of gene co-expression networks, in healthy mammary tissue and breast cancer samples. This could provide insight on the biological processes in which *PER3* is involved but also on the potential effects that alterations in its function could entail. Finally, we investigated the associations between *PER3* expression and disease-free survival in breast cancer for each specific intrinsic molecular subtype.

## Results

### Analysis of association between the *PER3*^VNTR^ polymorphism and cancer risk

The genotypes of the *PER3*^VNTR^ polymorphism were obtained for two independent cohorts of women derived from the Oslo University Hospital (Cohort 1) and the Netherlands Cancer Institute (Cohort 2). Cohort 1 included 1575 women diagnosed with breast cancer and 1640 controls whereas Cohort 2 comprised 560 cases and 567 controls (see Supplementary Table 1 available in Supplementary File 1). The observed genotype distributions did not present deviations from Hardy-Weinberg equilibrium for cases or controls in any of the cohorts (Cohort 1 cases: *p*-val = 0.89), (Cohort 1 controls: *p*-val = 0.82), (Cohort 2 cases: *p*-val = 0.98), and (Cohort 2 controls: *p*-val = 0.55).

Overall, unadjusted odds ratios showed a positive trend of association between breast cancer risk and *PER3*^VNTR^ long repeat allele, although it did not reach statistical significance under any tested model. We observed a non-significant slightly increased breast cancer risk associated with the homozygous 5-repeat allele (OR, 1.09; 95% CI, 0.85–1.40) and (OR, 1.37; 95% CI, 0.91–2.06) for cohorts 1 and 2, respectively. A non-significant positive association was also found for the heterozygous alleles (Cohort 1: OR, 1.05; 95% CI, 0.91–1.22), (Cohort 2: OR, 1.11; 95%CI, 0.87–1.42) and the combination of the 5-repeat variant alleles (heterozygous + homozygous) (Cohort 1: OR, 1.06; 95% CI, 0.92–1.22), (Cohort 2: OR, 1.15; 95%CI, 0.91–1.46) in both cohorts. Odds ratios and genotype frequencies from our data and previously published studies are shown in (Table 1).

**Table 1:** Genotype distributions of the different cohorts included in the meta-analysis.

Study	Genotype	Cases *N* (%)	Controls *N* (%)	OR (95% CI)
Cohort 1	4/4	761 (48.8%)	816 (49.7%)	1.00
	4/5	666 (42.2%)	679 (41.4%)	1.05 (0.91–1.22)
	5/5	148 (9.4%)	145 (8.9%)	1.09 (0.85–1.40)
	4/5 and 5/5	814 (51.7%)	824 (50.2%)	1.06 (0.92–1.22)
Cohort 2	4/4	245 (43.8%)	268 (47.3%)	1.00
	4/5	251 (44.8%)	248 (43.7%)	1.11 (0.87–1.42)
	5/5	64 (9.4%)	51 (8.9%)	1.37 (0.91–2.06)
	4/5 and 5/5	315 (51.7%)	299 (50.2%)	1.15 (0.91–1.46)
Zhu et al.	4/4	180 (46.0%)	206 (47.7%)	1.00
	4/5	175 (44.8%)	198 (45.8%)	1.01 (0.76–1.35)
	5/5	36 (9.2%)	28 (6.5%)	1.47 (0.86–2.51)
	4/5 and 5/5	211 (53.9%)	226 (52.3%)	1.07 (0.81–1.41)
Dai et al.	4/4	1092 (71.9%)	1181 (73.8%)	1.00
	4/5	406 (26.7%)	395 (24.7%)	1.11 (0.95–1.31)
	5/5	21 (1.4%)	24 (1.5%)	0.95 (0.52–1.71)
	4/5 and 5/5	427 (28.1%)	419 (26.2%)	1.10 (0.94–1.29)
Wirth et al.	4/4	85 (37%)	81 (38%)	1.00
	4/5	N.A.	N.A.	N.A.
	5/5	N.A.	N.A.	N.A.
	4/5 and 5/5	144 (63%)	131 (62%)	1.05 (0.71–1.54)

Odds ratios are provided for each cohort and comparison type (5/5 Vs 4/4. 4/5 Vs 4/4 and 5/5 plus 4/5 Vs 4/4). (N.A, data was not available for those genotypes)

Meta-analysis are known to increase statistical power and to provide better estimates of the effect sizes^18^, therefore we combined our data with results from previously published studies^15–17^ dedicated to examine the potential role of the *PER3*^VNTR^ polymorphism on breast cancer risk (Table 1). We calculated pooled odds ratio under fixed and random effect models by applying the inverse variance method. Overall, meta-analysis results showed a non-significant trend towards increased cancer risk in 5-allele repeat carriers. No differences were found when computing the pooled effect-sizes under fixed or random effect models due to the lack of heterogeneity. A non-significant 17% increase in breast cancer risk (OR = 1.17, 95% CI = 0.97–1.42) was observed for 5-allele repeat carriers under the homozygous model (5/5 Vs 4/4), whereas the heterogeneous (5/4 Vs 4/4) and dominant models (5/5 + 5/4 Vs 4/4) meta-analyses yielded non-significant increases of breast cancer risk of 9% (OR = 1.09, 95% CI = 0.99–1.18) and 7% (OR = 1.07, 95% CI = 0.98–1.18) in 5-allele repeat carries, respectively.

**Fig. 1:**
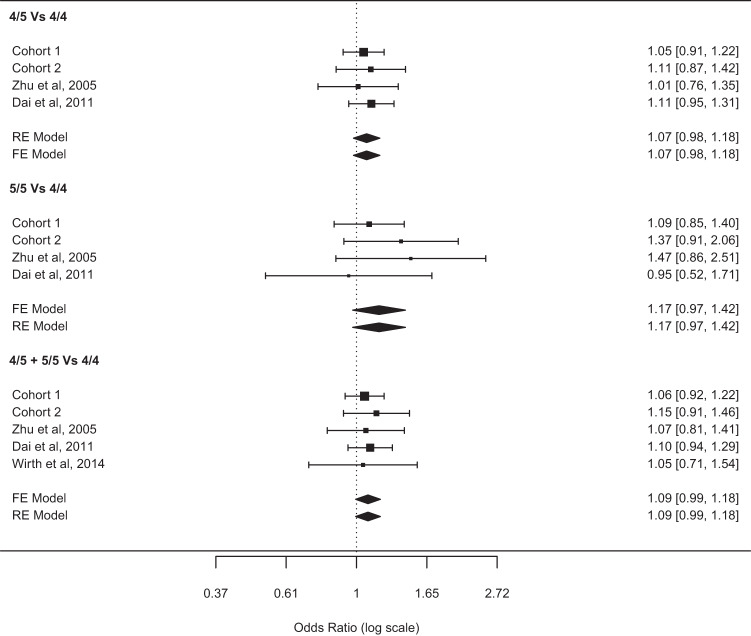
Meta-analysis results. Displaying pooled estimates obtained by combining information about C1 and C2 cohorts and Zhu, Dai and Wirth studies under the three different models (homozygous, heterogeneous, and dominant).

No significant between-study heterogeneity was observed under any tested model (Homozygous model, *I*² =0.0%, *Q* = 0.48, *p*-val = 0.92) (Heterogeneous model, *I*² =0.0%, *Q* = 2.07, *p*-val = 0.55), (Dominant model, *I*² 0.0%. *Q* = 0.45, *p*-val = 0.97). Figure 1 summarizes the meta-analysis results.

### Preferential allelic imbalance at the *PER3* locus in tumors and cell lines

**Fig. 2:**
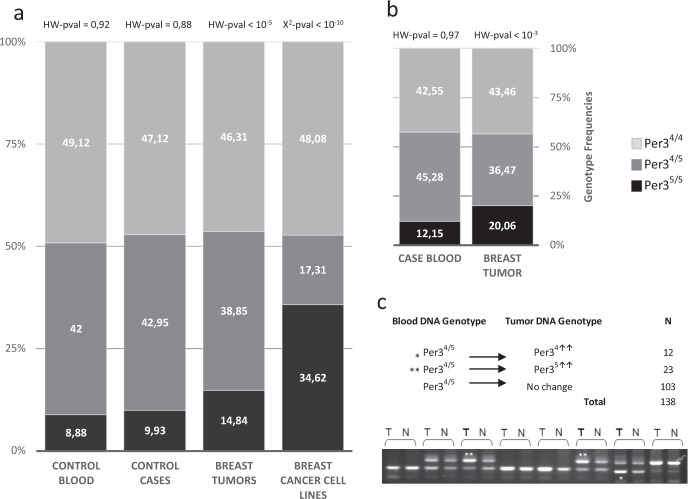
*PER3*^VNTR^ genotype distribution in human samples. **a***PER3*^VNTR^ genotype proportions in blood samples from control individuals in C1 and C2, blood samples from case individuals in C1 and C2, breast cancer tumor samples, and breast cancer cell lines. **b** *PER3*^VNTR^ genotype proportions of germinal (blood) and tumor samples of matched individuals. **c** Number of patients which presented heterozygous *PER3*^VNTR^ genotypes in blood samples (N) and homozygous *PER3*^VNTR^ genotypes in tumor samples (T). The image show the PCR analysis of genomic DNA of *PER3*^VNTR^ genotypes from matched tumor (T) and blood (N) samples of different individuals.

In addition to the case–control information, we obtained genotypes of 1304 breast tumor samples and 52 breast cancer cell lines. We observed an increase in the 5/5 genotype frequency from control blood samples compared to cell lines. 8.9% of control blood samples were 5/5, an increase of 1% in the 5/5 genotype frequency was found for case blood samples (9.9%). Tumors presented a 14.8% of frequency for the 5/5 genotype. Finally, cell lines showed the highest 5/5 genotype frequency with a 34.62%. This fact alongside with the loss of Hardy-Weinberg equilibrium in tumor samples and the significantly altered proportion of *PER3*^VNTR^ genotypes observed in cell lines ($X^2$ *p*-val = 8.64e-11) suggests that a selective pressure could be operating in favor of the 5/5 genotype acquisition during tumor development. Probably due to a 5-allele preferential retention after loss of heterozygosity (LOH) in region 1p36. Genotype frequencies for blood control and cases together with tumor samples and cell lines and the *p*-values for the Hardy-Weinberg and Chi-Squared tests can be found in Fig. 2a. The *PER3* genotypes for the 52 tested breast cancer cell lines and their molecular subtype classification based on different previously published works^19–23^ (See also https://lincs.hms.harvard.edu/about/approach/reagents/icbp43/), as well as the genotype distributions based on each classification are available at Supplementary Data 1. We analyzed and represented in bar graphs the frequencies of the different genotypes related with molecular subtypes. The frequencies of the PER3^5/5^ genotype were found to be higher in Basal-like and triple-negative breast cancers compared to other breast cancer subtypes, whereas PER3^4/4^ genotypes were more frequent on ER-positive, luminal and HER2-positive subtypes. However, the diverse genotype distributions observed in the distinct breast cancer subgroups did not reach statistical significance after Chi-squared analysis, most likely due to the low number of cell lines with molecular subtypes available for the analyses (*n* range 35–48) (Supplementary Data 1).

A subset of 329 samples for which paired blood and tumor samples were available was analyzed to determine if changes in the genotype were taking place in tumor samples compared to their blood counterpart. Overall, the 5/5 genotype was observed to be increased in tumors (OR, 1.61; 95% CI.1.02–2.56) (Fig. 2b), probably due to a reduction of germline heterozygous genotypes in the matched tumor samples. One-hundred and thirty-eight samples were originally heterozygotes in blood samples. Thirty-six out of the 138 (26%) samples, which were found to be heterozygotes in the blood, presented changes in genotype in their tumor counterpart. Twelve out of 36 (33.3%) presented LOH with retention of the 4-allele repeat in tumors whereas 24 out of 36 (66.6%) presented LOH with the permanence of the 5 repetition allele, showing a preferential shift towards the 5 repetition allele (Fisher’s Exact Test *p*-value = 0.0005). Binomial test *p*-values for the observed number of changes towards genotype 4 or 5 from blood to tumor samples were *p* = 0.98 and *p* = 0.03 respectively (see Fig. 2c). These results are compatible with a preferential allelic imbalance at the *PER3* locus in breast cancer in which the long allele repeat is preferentially retained.

### *PER3* co-expression structure in human and murine healthy mammary tissues

**Fig. 3:**
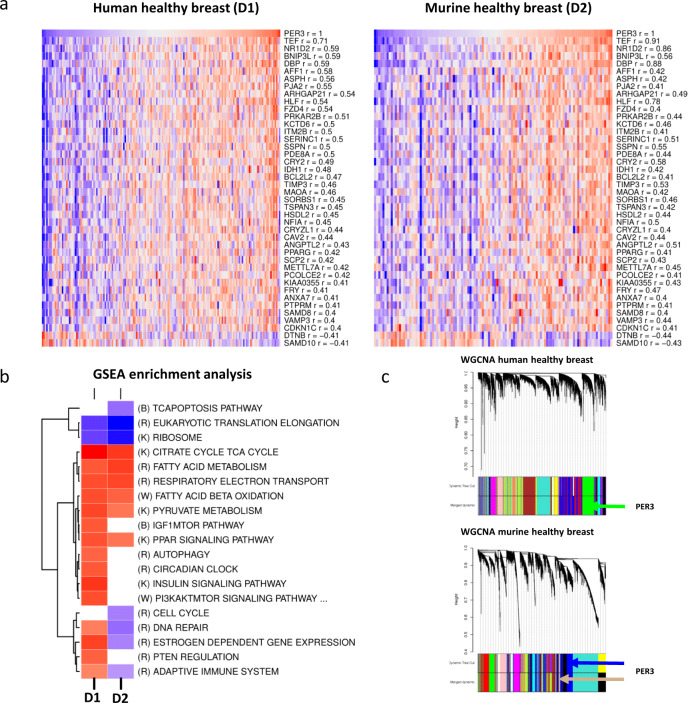
PER3 co-expression analyses in healthy human and murine mammary samples. **a** Genes showing absolute correlation values >0.4 in both human(D1) and murine (D2)healthy breast samples datasets. **b** Heatmap representing the GSEA enrichment analysis results in genes positively and negatively correlated with *PER3* in both human (D1) and murine (D2) healthy breast samples datasets. **c** Modules of co-expressed genes identified in D1 and D2 healthy breast cancer datasets by WGCNA.

Our data suggest that *PER3* alterations could play a significant role in breast cancer. To further characterize *PER3* functions in healthy mammary tissues we determined its co-expression structure using a human (D1) and a murine (D2) healthy mammary tissue gene expression datasets. For a detailed description of the datasets, see the Material and methods section and Supplementary Table 2. First, a robust list of *PER3* co-expression partners in healthy breast was obtained by retrieving those genes presenting absolute values of correlation with *PER3* higher than 0.4 in both D1 and D2. Genes in the list can be consulted in Fig. 3a and include several instances of genes previously linked to rhythmic processes including *CRY2*, *DBP*, *FZD4*, *HLF*, *NR1D2*, *PPARG*, and *TEF*^24^. Several *PER3* robust co-expression partners were also interesting given their potential implication in cancer related processes. For instance, *AFF1* has been linked to childhood lymphoblastic leukemia^25^ and *BCL2L2* and *BNIP2L* are related to cell survival control, and pro-apoptotic functions, respectively^26^. *CDKN1C* is an inhibitor of several G1 cyclin/CDK complexes which is involved in the regulation of several hallmarks of cancer, including cell proliferation, apoptosis, cell invasion and metastasis, tumor differentiation, and angiogenesis^27^, whereas *FRY* plays a role in centrosome integrity maintenance during mitosis and could interact with *AURKA* to mediate *PLK1* activation^28^.

Second, Gene Set Enrichment Analysis (GSEA) was carried out ordering all the studied genes by their correlation value with *PER3*. GSEA analysis results of the D1 dataset showed that *PER3* positively correlated genes were enriched in several biological processes besides circadian clock machinery (Fig. 3b) including pathways relevant for cancer such as, PI3K/AKT, insulin, and PPAR signaling, the synthesis of ATP thought the electron transport chain, the metabolism of fatty acids. *PER3* negatively correlated genes were enriched in peptide chain elongation, and cell–cell junction organization among others. Supplementary Table 3 shows the full GSEA enrichment results for D1 healthy mammary samples whereas Supplementary Fig. 1 shows GSEA plots of the top pathways enriched in genes positively and negatively correlated with *PER3* in D1. Murine data (D2) GSEA analysis results also suggested that genes positively correlated with *PER3* were enriched in pathways related to the oxidative phosphorylation, the metabolism of lipids and PPAR signaling, whereas genes negatively correlated with *PER3* in D2 were also enriched in biological processes linked to the translation. In addition, genes negatively correlated with *PER3* in D2 were also enriched in cell cycle, DNA damage, and apoptosis pathways (Fig. 3b).

Supplementary Table 4 shows the full GSEA enrichment analysis results for D2 and Supplementary Fig. 2 depicts GSEA plots for a selection of the top associated pathways linked to both positively and negatively *PER3* co-expressed genes in D2.

Finally, weighted gene co-expression network analyses (WGCNA) identified 20 and 19 modules of co-expressed genes in the D1 and D2 human and murine healthy breast datasets, respectively (Fig. 3c). Supplementary Fig. 3 show the power selection plots for the construction of the adjacency matrix, the dendrogram depicting the co-expression modules detected by WGCNA, and the correlation between the eigengenes of each detected module for D1 and D2. In the case of D1, *PER3* was found to be placed in the green module which was heavily enriched in adipocyte, endothelial cells, and smooth muscle cells genetic markers (*p*-adj = 1.56e-23, 2.00e-31, and 9.84e-09). GO enrichment analysis showed that green module genes were enriched in functional categories related to cell adhesion, response to endogenous stimulus, circulatory system development, cell motility, regulation of cell proliferation, and lipid metabolism. The full cell type specific markers and GO enrichment results for the D1 healthy tissue green module can be checked in Supplementary Fig. 4 and Supplementary File 2. *PER3* was found to be placed in the D2_tan module which did not present enrichment in biological processes or cell type specific markers, however, the D2_tan module presented an eigengene correlation of 0.77 with the D2_blue module which was also found to be enriched in adipocyte (*p*-adj: 9.65e-12) and endothelial cells (*p*-adj = 1.10e-12) specific cell type markers. The full cell type specific markers and GO enrichment results for the D2 healthy tissue green module can be checked in Supplementary Fig. 5 and Supplementary File 3.

### *PER3* differential co-expression between intrinsic breast cancer subtypes and healthy mammary tissues

To determine the co-expression changes observed between *PER3* healthy mammary tissues and the different tumor subtypes defined by the PAM50 algorithm we performed differential co-expression analysis in the human dataset (D1). The complete D1 gene expression dataset and the intrinsic subtype breast cancer classification of the included samples are available at the following link: https://osf.io/azgby/. Three hundred and twenty genes showed significant differential co-expression with *PER3* in the healthy tissue versus luminal A (LumA) breast cancer analysis using D1 data. Most of the genes (275) presented positive correlations with *PER3* in the healthy samples and lost this correlation in LumA cancer. gProfileR enrichment analysis showed enrichment in KEGG functional categories mainly related to Metabolic pathways (*p*-adj: 3.531 × 10^−7^) including the citrate cycle (TCA cycle), Fatty acid metabolism, pyruvate metabolism, and AMPK signaling (*p*-adj: 1.860 × 10^−6^, 3.571 × 10^−4^,3.007 × 10^−3^, and 1.071 × 10^−2^). Analogous instances of REACTOME pathways were also found. Similar results were found when computing *PER3* differential co-expression between the healthy mammary tissue and luminal B (LumB) tumors. In this case 607 genes were found to be differentially co-expressed with *PER3* (FDR < 0.05) from which most 510 presented positive correlations with *PER3* in the healthy mammary tissues and lost its correlation or even acquired negative correlation with *PER3* in LumB tumors. Overrepresentation analysis also showed enrichment in KEGG and REACTOME pathways linked to the Citrate cycle (TCA cycle), AMPK signaling, and the metabolism of lipids (*p*-adj = 4.967 × 10^−7^, 7.440 × 10^−4^, 4.510 × 10^−4^).

D1 *PER3* differential co-expression between healthy mammary tissues and basal breast cancers yielded five hundred and twenty-one differentially co-expressed genes with *PER3*. In this case, however, functional categories related to the ATP synthesis through the TCA cycle were not found to be enriched whereas light enrichment in some gene ontology gene sets related to lipid metabolism were found (*p*-adj = 4.035 × 10^−2^).

Finally, D1 differential co-expression between healthy breast tissues and Her2 tumor samples yielded five hundred and twenty differentially correlated genes, presenting similar enrichment results that those found in the Healthy mammary tissue versus LumA and LumB analysis. Overall changes in *PER3* co-expression between healthy mammary tissues and breast cancer were related to energy and lipid metabolism. Supplementary Data 2 includes the genes showing patterns of significant differential co-expression with *PER3* in all the intrinsic breast cancer subtypes compared to healthy breast tissues, as well as their overrepresentation enrichment analysis results in biological processes.

### *PER3* differential co-expression between breast cancer intrinsic subtypes

**Fig. 4:**
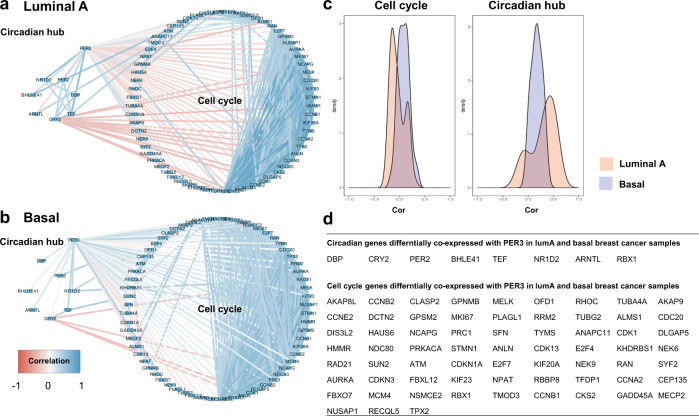
Differences in *PER3* co-expression structure between luminal A and basal breast cancer samples. **a***PER3* co-expression network in lumninal A breast cancer samples including cell cycle and circadian hub genes found to be differentially co-expressed in luminal A and basal breast cancer samples. **b** *PER3* co-expression network in basal breast cancer samples including cell cycle and circadian hub genes found to be differentially co-expressed in luminal A and basal breast cancer samples. **c** Densities of the correlations between *PER3* and both the eight circadian related genes found to be differentially co-expressed between the LumA and the basal subtypes (left plot) and the 66 cell cycle genes found to be differentially co-expressed between LumA and Basal (right plot). **d** Table depicting the list of genes linked to the circadian hub and the cell cycle found to be differentially correlated with PER3 in luminal A and basal breast cancer samples.

To determine the changes in *PER3* co-expression structure between the different breast cancer subtypes, differential co-expression analysis of *PER3* was performed between breast cancer subtypes classified using the PAM50 algorithm implemented in the genefu package. *PER3* differential co-expression analysis between LumA and basal array breast cancer samples yielded 556 differentially co-expressed genes. Enrichment analysis showed that genes differentially co-expressed with *PER3*, in LumA and Basal cancer samples, were mainly linked to biological functions involved in cell cycle, DNA damage response, ATP synthesis, and circadian rhythms, including instances of the gene ontology biological process branch (mitotic cell cycle process, regulation of mitotic cell cycle, mitotic G1 DNA damage checkpoint, signal transduction by p53 class mediator, mitochondrial ATP synthesis coupled electron transport, *p*-adj = 5.84 × 10^−10^, 8.17 × 10^−7^, 4.2 × 10^−3^, 2.436 × 10^−2^), and kegg pathways including KEGG’s Cell cycle (*p*-adj = 3.755 × 10^−5^), p53 signaling pathway (*p*-adj = 8.928 × 10^−3^) and circadian rhythm (*p*-adj = 3.808 × 10^−2^). Several circadian genes were found in the list of significantly differentially co-expressed genes, some of them presented positive co-expression with *PER3* in the LumA breast cancers (DBP, CRY2, PER2, BHLHE41, TEF, and NR1D2) and a loss or a significant reduction of the correlation values was observed in the basal subtype, whereas two circadian genes (ARNTL and RBX1) presented negative or null correlations with *PER3* in LumA samples and positive correlations in the basal subtype. Sixty-six genes which belonged to (GO:1903047, mitotic cell cycle process) were found to be differentially co-expressed with *PER3*. Fifty of them presented lower values of correlation with *PER3* in the LumA subtype compared to the basal type. Figure 4 show the changes in the *PER3* co-expression structure of circadian and cell-cycle related genes in the comparisons between Luminal A and Basal breast cancer samples.

*PER3* differential co-expression analysis between LumB and basal samples yielded 910 differentially co-expressed genes however, analysis results did not show strong enrichment results in any functional category. Finally, only 25 *PER3* differentially correlated genes were found when comparing Her2 with basal samples. No pathway enrichment was found for this set of differentially co-expressed genes. The complete list of differentially co-expressed genes for each comparison and the overrepresentation analysis of the significant differentially co-expressed genes can be found in Supplementary Data 2.

### Disease-free survival analysis based on expression status of *PER3* and its robust co-expression partners

Prior studies carried out by our group determined that low *PER3* expression was linked to worse disease-free survival in estrogen receptor (ER) positive and LumA tumors and was not related to changes in survival in ER negative and basal tumors. To validate this association, we performed logrank tests using relapse-free survival (RSF) data from Kaplan–Meier plotter (KMplotter) for all breast cancer samples and each intrinsic breast cancer subtype independently. The analyses were carried out for *PER3* and the complete set of *PER3* robust co-expression partners identified in the human and murine healthy breast tissue analyses. In addition, an average expression signature was constructed using the expression values of those *PER3* co-expression partners that were found to be associated with a significant increase or reduction in relapse-free survival in the complete breast cancer dataset. Then, the average expression signature was tested for association with relapse-free survival.

**Fig. 5:**
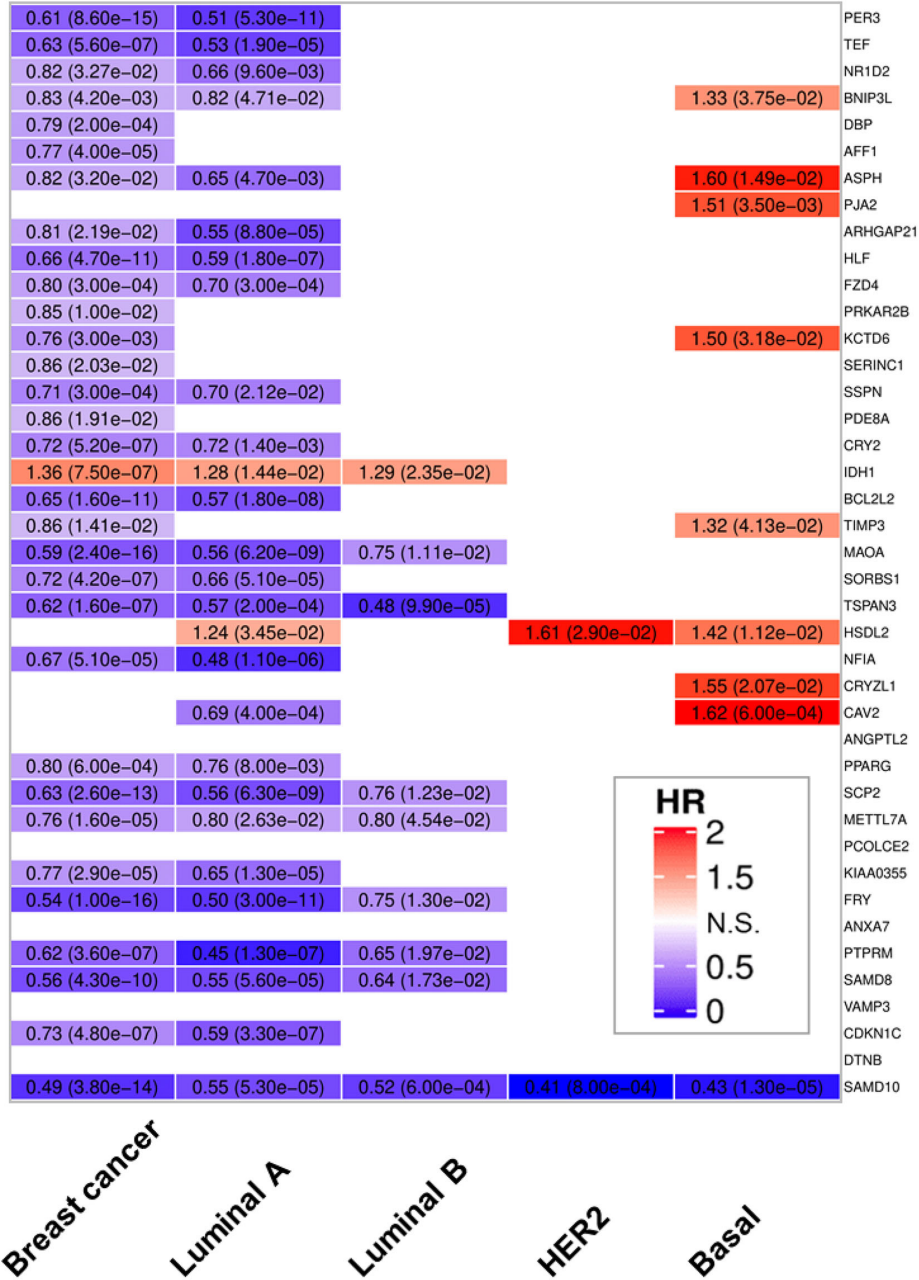
Survival analysis. Logrank test analysis results showing the associations of the expression levels of *PER3* and its robust co-expression partners with relapse-free survival in samples derived from all breast cancer subtypes and each intrinsic breast cancer subtype (i.e., luminal A, luminal B, HER2, and basal). Each cell shows the Hazard Ratios (HR) and the *p*-values derived from the analyses. Cell color intensities are proportional to HR values. Blue hues indicate genes for which low expression levels are associated with shorter relapse-free survival times, whereas red hues indicate genes for which high expression levels are associated with shorter relapse-free survival times. Non-colored cells indicate no association between gene expression and RFS.

**Fig. 6:**
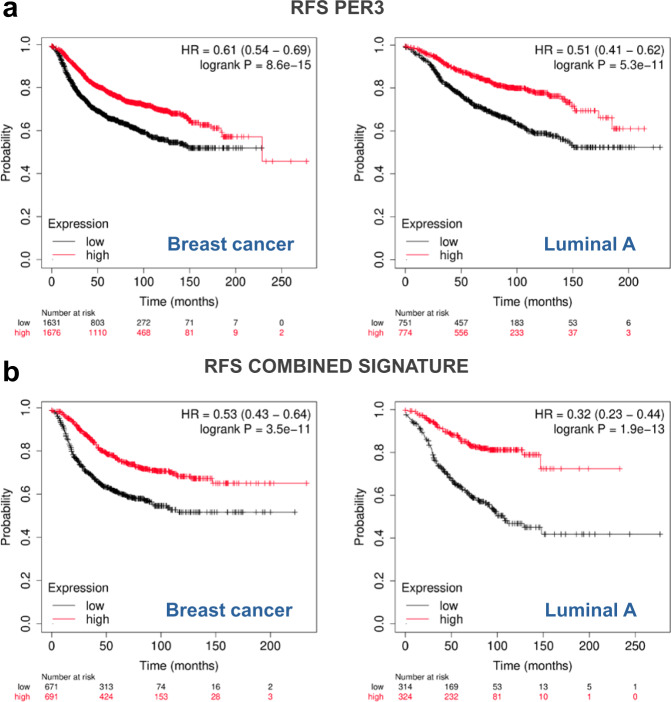
Kaplan–Meier curves depicting RFS analyses results. **a** RFS analysis results based on *PER3* expression levels using the complete breast cancer dataset (left panel) and the subset of samples classified as Luminal A (right panel). **b** RFS analysis results based on the average gene expression of genes significantly co-expressed with *PER3* in human and murine healthy mammary tissues that were significantly associated with RFS in univariate analysis. The left panel shows the results for the complete breast cancer dataset whereas the right panel does it for the luminal A subset. Red lines represent samples showing high expression values (first tertile) of *PER3* or the combined signature whereas black lines represent low expression values (third tertile).

In the case of *PER3*, the logrank tests were carried out using 3307, 1525, 1000, 210, and 568 samples for the complete breast cancer dataset and the lumA, lumB, Her2, and basal analyses, respectively. Low values of *PER3* expression were found to be significantly associated to worse relapse-free survival outcomes in both, the complete dataset (HR = 0.61, *p*-val = 8.6 × 10^−15^) and in LumA samples (HR = 0.51, *p*-val = 5.3 × 10^−11^) but not in the rest of breast cancer subtypes. The expression levels of 32 genes that showed significant co-expression patterns with *PER3* in human and murine healthy mammary tissues were significantly associated to relapse-free survival in the complete breast cancer dataset whereas 27 did it in lumA samples. *TEF*, *HLF*, *BCL2L2*, *MAOA*, *SCP2*, *FRY*, *PTPRM*, and *CDKN1C1* were among the top associated genes for which low expression values were significantly linked to poor relapse-free survival outcomes in both the complete breast cancer dataset and the lumA subset. Figure 5 shows the significant relapse-free survival analysis results for all the tested genes in the complete breast cancer dataset and for each intrinsic breast cancer subtypes. Figure 6a show the Kaplan–Meier curves based on *PER3* expression for the complete breast cancer dataset and the subset of samples classified as luminal A.

Finally, for those groups of samples in which *PER3* expression was significantly associated with RFS (the complete breast cancer dataset and the luminal A subset of samples) an average gene expression profile including the expression levels of all genes significantly associated with RFS in univariate analysis was constructed. Then, the average profile was tested for association with RFS. In the case the complete set of breast cancer samples low expression levels of the combined signature were associated with reduced relapse-free survival (HR = 0.53, *p*-val = 3.5 × 10^−11^), the same pattern was observed in luminal A breast tumors (HR = 0.32, *p*-val = 1.9 × 10^−11^) (Fig. 6b). Supplementary Table 6 shows the list of genes and array probes used to construct the average gene expression profiles for the complete set of breast cancer samples and the luminal A subset. Our analyses suggest that changes in gene expression in *PER3* and its co-expression partners are associated with relapse-free survival in overall and more specifically in luminal A breast cancers.

Survival and differential co-expression data suggest that *PER3* could be important modulating the relapse-free survival outcomes of LumA breast cancer patients by regulating cell cycle though mechanisms not fully elucidated that may involve loss of cell cycle control though decoupling of circadian function.

## Discussion

Our data on this combined germline and somatic genetic analysis of associations between the *PER3* polymorphism and breast cancer suggest that the long repeat *PER3*^*VNTR*^ allele may influence breast cancer at two different levels. First, despite not reaching significance, a trend towards association of the long allele repeat (5/5) with an increased risk of suffering breast cancer was observed using a meta-analytical approach. A non-significant increase in risk was also observed in the individual analysis of both of our cohorts, which to our knowledge, represent the biggest individual *PER3*^*VNTR*^ and breast cancer association study to the date.

The Light at night (LAN) hypothesis states that the exposure to visible light during night, lowers the nocturnal melatonin production by the pineal gland which in turn increases the risk of cancer development due to melatonin anti-proliferative effects and melatonin enhancement of the immune system^29–32^. Predictions of this hypothesis which include among others (increased breast cancer risk among non-day shift workers, blindness lower risk, and co-distribution between population level community nighttime and breast cancer incidence)^29,30^ are supported by increasing evidence. For example clinical studies have demonstrated a significant decrease in the peak concentrations of melatonin in women with metastatic cancer^33^. Furthermore, blind women unable to detect the presence of environmental light and hence showing no daily melatonin levels reduction are at lower risk of breast cancer diagnosis than blind women who perceive light and have daily decreases in melatonin levels^34^. Melatonin levels have been linked to an increase of cancer risk and tumor growth by several experimental studies^35^. In addition, two major reviews of the literature concluded that long-term exposure to night-shift work increases risk for breast cancer^36,37^. Experimental approaches in murine models have confirmed that LAN markedly increases the growth of human breast cancer xenografts in rats^30^. There is growing evidence pointing out at *PER3*^*VNTR*^ polymorphism as a possible modulator of some of the processes previously described. For example, it has been reported that *PER3* levels correlate significantly with sleep-wake timing and the timing of melatonin and cortisol, being this correlation stronger for the 5/5 individuals^38^. Moreover, non-visual light responses at the short-wavelength range such as melatonin concentration reduction are thought to be modulated by *PER3* in a polymorphism dependent fashion. In particular it has been observed that blue-enriched light induced a significant suppression of the evening rise in endogenous melatonin levels in *PER3* (5/5) individuals but not in *PER3* (4/4)^39^.

Besides the *PER3*^*VNTR*^ and cancer associations framed inside LAN hypothesis another avenues of association between cancer and *PER3* have been explored. For instance, *PER3*^*VNTR*^ polymorphism has been related with a modulation of the sympathovagal balance under sleep deprivation conditions, with 5/5 individuals, showing a higher sympathetic predominance under this conditions^40^. It has also been reported that noradrenaline, the postsynaptic neurotransmitter of the sympathetic central nervous system, has a stimulatory effect over cell proliferation and migration and tumor progression^41^.

Awakening cortisol levels have been found to be higher in individuals with 4/5 or 5/5 genotype compared with those with 4/4 genotypes and those differences were stronger when the subset which worked more afternoon or night shifts was analyzed^42^. It is important to notice that some of the phenotypical allele dependent manifestations of *PER3* are conditional in nature and only manifest in specific situations such as when altered sleep patterns are present. Besides the aforementioned other genotype dependent effects under sleep deprivation conditions have been reported. For example, attentional performance impairment is greater in *PER3* 5/5 individuals under sleep deprivation conditions^43^. Murine models of humanized *PER3* 5/5 allele mice have also shown a modified homeostatic response for individuals under sleep deprivation conditions^44^. The homozygous 5/5 genotype was found to be the genotype with the lowest frequency in all the analyzed cohorts. In cohorts 1 and 2 the proportion of individuals carrying this genotype were ~9% which is compatible with the proportions observed by Zhu and collaborators, however in Dai’s study, 5/5 carriers represented only about a 1.5% studied population which raises questions about the genotype distribution in different human populations which should be object of further research. Rare and low frequency variants could explain additional disease risk or trait variability^45^ and rare genetic variants of *PER3* have been found previously significantly associated with a number of mood disorders features^46^. These facts taken together with the trend of risk increase observed in our meta-analysis for the 5-repeat allele carriers suggests that that *PER3* long allele repeat could be increasing the breast cancer risk of a subset of patients exposed to specific environmental conditions. In particular, a subgroup characterized by altered sleep patterns or more exposed to LAN effects. Further research is needed to assess the interactions between the *PER3*^*VNTR*^ polymorphism and sleep disruption and its link with breast cancer risk.

The second level of association between *PER3*^*VNTR*^ and Breast Cancer is related with the cell-autonomous behavior within the tumor cells. This idea is supported by our data showing a preferential allelic imbalance at the *PER3* locus and by the association found between low *PER3* expression levels and worse disease-free survival outcomes in Luminal A breast cancers. Combined data of patient samples and cell lines suggest that breast tumors that undergo genetic alterations on chromosome region 1p36 preferentially lose the more common 4 repeat allele, and retain the *PER3* 5 repeat allele. This preferential retention could be due to the fact that changes in *PER3* function derived from the presence of an additional VNTR repetition have a beneficial effect on tumor fitness. Further studies are necessary to elucidate the mechanism by which the change in genotype takes place which could imply loss of heterozygosity, mitotic recombination leading to homozygosity, or chromosomal non-disjunction.

Altogether our data suggest that *PER3* 5/5 allele is preferentially selected during tumor development. It still has to be elucidated if *PER3* 5/5 allele confers a selective advantage during development. However, our co-expression analysis suggests that *PER3* could be involved in several molecular mechanisms related to cancer which include energy metabolism, signaling through cancer related pathways such insulin and PI3K/AKT signaling and cell cycle control. Extensive evidence exists linking this processes to cancer in general and breast cancer in particular^47–55^. For instance, PTEN an important negative regulator of the PI3K/AKT signaling pathways, which over activity leads to cell growth and tumor proliferation playing also an important role in endocrine resistance in breast cancer^56^, is involved in breast tumorigenesis and tumor progression and reduced expression of this gene in mammary tumor samples has been linked to a bigger tumors and higher pathological stages and the expression of estrogen receptor(ER) and the progesterone receptor (PR)^57^. IGF1 also plays a central role in cancer development, stimulates mitosis, and inhibits apoptosis. In particular, for breast cancer odds ratio for women in the highest versus the lowest fifth of IFG1 serum concentration was 1.28 (95% CI 1.14–1.44; *p* < 0.0001) this association was not altered by adjusting for IFGBP3^58^. Polymorphisms of circadian genes are associated with serum hormone levels, Importantly, the effect of *PER3*^*VNTR*^ polymorphism in IGF1 serum levels has been studied concluding that *PER3* longer allele carriers presents higher serum levels of IGF1 and IGF1 to IGFBP3 ratios^59^. This axis has also been associated with tumor growth acceleration through LAN exposure, in particular a continuous activation of IGF1-1R/PDK1 signaling after LAN exposure have been reported in human breast cancer xenografts^60^. Moreover, several studies have linked some of the pathway enriched in PER3 co-expressed genes together. The central circadian clock has been reported to be a key regulator of the energy metabolism^61^ as well as the PI3K/AKT signaling axis which works as a master regulator of aerobic glycolytic metabolism and it is also involved in the regulation of the oxidative metabolism^62^.

We have shown that low levels of both *PER3* expression and its robust co-expression partners are associated with a reduction in relapse-free survival in Luminal A breast cancer but not in other subsets of patients. Differential co-expression analysis of *PER3* between breast cancer subtypes suggest that *PER3* present modest negative correlation with many cell cycle related genes in Luminal A samples but not in the other subtypes, especially in the basal subtype. This suggest that *PER3* could be implicated in the regulation of the cell cycle in Luminal samples and could explain why low expression levels of *PER3* are associated with decreased disease-free survival in this particular subtype. More research will be needed to address many of the reported observations and to evaluate the functional impact of *PER3*^*VNTR*^.

Finally, we present our study as the biggest case–control study examining *PER3*^*VNTR*^ and breast cancer associations to the date. Nevertheless, it would be suitable to significantly increase the number of samples to make it comparable to the state-of-the-art variant-trait association studies such as GWAS. It is also expected to enroll more cases including information about the breast cancer subtype, which will enable the examination of the effect of the polymorphism in each specific subtype. The link between the polymorphism and its potential effect on *PER3* expression and function, that could not be examined yet given the nature of our data, should be object of future research.

## Methods

### Study population

This study utilized samples from previously published studies^20,61,63–72^ and does not include any novel samples. Blood samples for this study were obtained from the laboratories of two independent groups of cases and controls, Oslo University Hospital^61,63–66^ and Netherlands Cancer Institute^67–69^. The Oslo University Hospital cohort, henceforth (cohort 1, C1), included 1575 cases and 1640 controls; whereas, the Netherlands Cancer Institute cohort, hereafter (cohort 2, C2), comprised 560 cases and 567 controls. Genotypes derived from 1304 breast tumor samples were also obtained from these cohorts (C1 and C2). Additionally, tumor samples from a third cohort (C3) were also included from the Clinical Hospital of the University of Valencia^70,71^. (Supplementary Table 1). Genotypes for matched germinal (blood) and tumor samples were generated for a subset of 329 patients. Finally, a collection of 52 breast cancer cell lines^20,72^ was also genotyped. All samples used in this study were anonymized before we obtained the samples, and contained no personal or clinical data other than tissue origin (blood for cases/controls and tumors). All participants for these studies provided informed consent for the use of these sample for future research purposes. Since this retrospective study only used tissue samples from previously published studies, and did not include any clinical or personal data, additional ethics approval and informed consent was not required according to the country of origin guidelines.

### *PER3* genotyping

Genomic DNA was extracted using standard procedures^73^. The genotype for the VNTR polymorphism was determined by PCR assay. PCR primers used were 5′-TGGCAGTGAGAGCAGTCCT-3′ (forward) and 5′- AGTGGCAGTAGGATGGGATG-3′ (reverse). The PCR was performed in a reaction mixture of 10 µL containing 1 µL of DNA from a (10 ng/µL) solution, 0.4 µL of each primer, 0.5 µL of MgCl2M, 0.4 µL of each dNTP, 0.4 μL of Taq Gold polymerase (ROCHE), and 5.9 μL H2O. The PCR cycling conditions were 10 min at 94 °C followed by 35 cycles of 10 s at 94 °C, 30 s at 68 °C, and 30 s at 72 °C, with a final step at 72 °C for 3 min. PCR products were resolved and separated in a 1.6% Electrophoretic gel (Lonza). After electrophoresis homozygous for the 5 repeats allele were observed as a 257-bp DNA band. Homozygous alleles with 4 repeats were represented by a single 193 bp DNA band. Heterozygotes showed both bands in the gel. Gels were analyzed by 3 different researchers blinded to samples IDs. Samples from Cohort 2 were genotyped as follows: the primer sequence and PCR conditions were the same than previous cohorts, but forward primer was fluorescently labeled (FAM or VIC) for the genotyping. The PCR products were analyzed on the “ABI PRISM 3730 DNA analyzer” and the results were analyzed using the GeneMapper Software (Life Technologies) instead of visualizing by electrophoresis gel. Several samples were genotyped by both systems to double-check the genotypes.

### Statistical analysis and meta-analysis methods

Odds ratios were computed for homozygous (5/5 Vs 4/4), dominant (5/4 Vs 4/4), and heterogeneous (5/5 + 5/4 Vs 4/4) models for both cohorts (C1 and C2) using R statistical programming language. Meta-analysis under fixed and random effect models were carried out using the R metafor package (10.18637/jss.v036.i03).

### Gene expression datasets and array data preprocessing

Two datasets were used for the analysis of the co-expression structure of *PER3*. The first dataset (D1) was constructed combining studies found in Gene Expression Omnibus (GEO, https://www.ncbi.nlm.nih.gov/geo/) which included breast cancer and healthy breast samples analyzed with the Affymetrix platform hgu133plus2. D1 contained 167 healthy breast tissue, 1253 Luminal A (LumA), 1379 Luminal B (LumB), 639 Her2, and 1175 basal subtype breast cancer samples. Supplementary Table 2 details the studies included in the D1 dataset. Briefly, to generate the D1 dataset, raw data from each individual study (CEL files) was downloaded from GEO and the oligo^74^ and affy^75^ packages were used to read them and perform normalization and summarization using the RMA method, which was followed by quantile between-sample normalization and log2 transformation. Probes targeting the same gene were collapsed using the collapseRows function from the WGCNA^76,77^ package selecting the MaxMean method. For each study, the breast cancer samples were classified using the PAM50 algorithm included in the genefu package^78^. The combat function included in the SVA package^77^ was then used to remove batch effects taking into account the information regarding to the tumor subtype. Finally, all data was combined in a single matrix.

The second dataset (D2) was obtained from a mouse healthy mammary tissue study placed at GEO under the GSE46077 accession number and was carried out using the Affymetrix Mouse Gene 1.1 ST. Array platform, which included 115 samples. Normalization was carried out following the same methodology used for D1.

### Healthy breast tissue co-expression analysis

The *PER3* co-expression structure in healthy mammary tissue was evaluated using the healthy breast tissue samples available at each dataset. Spearman correlations were computed between *PER3* and all other genes. A robust list of co-expressed genes was derived by selecting those genes showing absolute values of correlation with *PER3* higher than 0.4 in both datasets. The resulting list was tested for functional enrichment using the GprofileR (https://biit.cs.ut.ee/gprofiler/) web tool and using Gene Set Enrichment Analysis (GSEA, http://software.broadinstitute.org/gsea/index.jsp).

### Differential co-expression analysis

Differential co-expression was carried out as follows: First, gene expression correlations were computed between *PER3* and all the other genes. When comparing to groups (i.e. healthy breast versus LumA) both correlation vectors were transformed following the Fisher’s method, Eq. (1).

1 $$Z = 0.5 \times \log \frac{{1 + r}}{{1 - r}}$$

When comparing both groups the differences between the *z* values were computed, Eq. (2):

2 $$Z_{{\mathrm{Diff}}} = Z_1 - Z_2$$

Then the standard deviations of the differences were obtained from the following expression, Eq. (3):

3 $$Z_{{\mathrm{DiffSD}}} = \sqrt {\frac{1}{{\left( {N_1 - 3} \right)}} + \frac{1}{{\left( {N_2 - 3} \right)}}}$$

where $N_1$and $N_2$are the number of samples used to computed the correlations in group a and b respectively. Finally, the ratio between $Z_{{\mathrm{Diff}}}$and$Z_{{\mathrm{DiffSD}}}$ was computed, and the significance of the test was assessed using the normal distribution. The retrieved *p*-values were then corrected for multiple comparisons using the false discovery rate (FDR) method.

### WGCNA analysis

To determine the placement of *PER3* genes in the context of the whole gene co-expression network structure of mammary healthy tissues, we constructed unsigned gene co-expression networks using the WGCNA package^79^. A threshold fit of 0.75 was selected and the deep split parameter was set to 3. Modules were then enriched in functional categories using two gene set sources, Reactome and the biological processes branch of Gene Ontology (GO). Cell type marker enrichment analysis was carried out using the PANGAO database (https://panglaodb.se/markers.html#) and hypergeometric tests to assess for significance.

### Relapse-free survival analysis based on the expression status of *PER3* and its robust co-expression partners

To determine if the expression levels of *PER3* and its co-expression partners were associated with disease-free survival in breast cancer we used KMplotter^80^. This online tool includes relapse-free survival (RFS) data for a subset of the studies included in our D1 gene expression dataset. For the complete breast cancer datasets and each molecular breast cancer subtype (LumA, LumB, Her2, and Basal-like) we extracted survival information based on the expression status of *PER3*. *PER3* expression levels were trichotomized and the disease-free survival of patients displaying *PER3* expression levels in the first (high expression) tertile was compared to the DFS of patients displaying *PER3* expression in the third tertile (low expression) by means of logrank tests. The same procedure was carried out in order to determine the association between the levels of expression of those genes found to be significantly co-expressed with *PER3 (*in both human and murine healthy breast tissues) with relapse-free survival. In the case of the complete breast cancer dataset and the luminal A subset of samples, an average gene expression profile including the expression levels of all genes significantly associated with RFS in univariate analysis was constructed. Then, the average profile was tested for association with RFS.

### Functional enrichment

Functional enrichment analysis was carried out using two different strategies. Gene Set Enrichment Analysis (GSEA) was carried out to determine functional enrichment of *PER3* co-expressed genes using the fgesea package^81^. Overrepresentation analysis was carried out using the g:Profiler online tool (https://biit.cs.ut.ee/gprofiler/gost).

### Reporting summary

Further information on research design is available in the Nature Research Reporting Summary linked to this article.

## Supplementary information

Supplementary Information

Reporting Summary

Supplementary Data 1

Supplementary Data 2

## Data Availability

All data generated during this study are included in this published article (and its supplementary information files). The data analyzed and not included in this paper are available on Gene Expression Omnibus repository (GEO, at https://www.ncbi.nlm.nih.gov/geo/). The GEO identifiers for the datasets analyzed are available on Supplementary Table 2 in the Supplementary File 1. Supplementary files 2 and 3 are available at https://osf.io/azgby/.
