# Supplementary material for: Circadian PERformance in breast cancer: a germline and somatic genetic study of PER3VNTR polymorphisms and gene co-expression
Source: NPJ Breast Cancer. 2021 Sep 10;7:118. doi: 10.1038/s41523-021-00329-2 (PMC8433453; doi:10.1038/s41523-021-00329-2)
Supplement: Supplementary file 1 — Supplementary Information [file 41523_2021_329_MOESM1_ESM.pdf]

Supplementary data

Summary of contents:

1) Supplementary table 1: Samples (cases, controls and tumor DNAs) from three different cohorts.

1) Supplementary table 2: GEO accession numbers and sample distribution of the studies selected to construct the D1 dataset.

2) Supplementary table 3: Gene Set Enrichment Analysis (GSEA) results of PER3 co-expression in D1 healthy human mammary tissues.

3) Supplementary figure 1: Selection of top pathways enrichen in both positively and negatively PER3 co-expressed genes in the healthy breast tissue D1 dataset.

4) Supplementary table 4: Gene Set Enrichment Analysis (GSEA) results of PER3 co-expression in D2 healthy murine mammary tissues.

5) Supplementary figure 2: Selection of top pathways enrichen in both positively and negatively PER3 co-expressed genes in the healthy breast murine tissue D2 dataset.

6) Supplementary table 5: Pathways jointly associated to PER3 co-expressed genes in D1 and D2.

Pathways jointly associated to PER3 positively correlated genes in D1 and D2 included TCA cycle, fatty acid metabolism, PPAR signaling, TGFB signaling, insulin receptor signaling among others. Instances of PI3K signaling pathways were also found to be enriched in PER3 positively co-expressed genes in both datasets. Pathways jointly associated to negatively correlated genes in D1, D2 included immune system, and translation related pathways

7) Supplementary figure 3: WGCA plots for healthy human mammary tissues D1.

D1 healthy breast tissues WGCA analysis produced 20 modules of co-expressed genes. Supplementary figure 3 A, B, and C show information about the D1 WGCA analysis results. PER3 was placed in the green module which was heavily enriched in adipocyte, endothelial cells, and smooth muscle cells genetic markers (p-adj = 1.56e-23, 2.00e-31, and 9.84e-09). GO enrichment analysis and REVIGO summarization showed that green module genes were enriched in functional categories related to cell adhesion, response to endogenous stimulus, circulatory system development, cell motility, regulation of cell proliferation, and lipid metabolism.

8) Supplementary figure 4: Enrichment in PanglaoDB cell type specific markers in the D1 human healthy mammary tissue dataset.

9) Supplementary figure 5: Enrichment in PanglaoDB cell type specific markers in the D2 murine healthy mammary tissue dataset.

D2 healthy murine breast analysis yielded 19 co-expression modules. Supplementary figure 3 D, E, and F shows information about the D2 WGCA analysis results. PER3 was found to be placed in the D2_tan module which did not present enrichment in biological processes or cell type specific

markers. Then we search for the modules showing the highest correlation values with the D2_tan module eigenge. The D2_blue module presented an eigengen correlation of 0.77 with the D2_tan module and was heavily enriched in Adipocyte (p-adj: 9.65e-12), Endothelial cells (p-adj = 1.10e-12), and Loop of Henle cells (p-adj = 7.36e-11) specific cell type markers.

10) Supplementary Table 6: Genes and probes used to construct the average gene expression profiles for relapse-free survival analysis in the complete breast cancer dataset and the luminal A breast cancer analysis.

Sample Cohorts	Origin	Institution	Controls	Cases	Tumor	Total
**Cohort 1 (C1)**	OSLO	*Institute for Cancer Research, Oslo University Hospital The Norwegian Radiumhospital*	1640	1575	397	3612
**Cohort 2 (C2)**	AMSTERDAM	*The Netherlands Cancer Institute. Antoni van Leeuwenhoek Hospital*	567	560	184	1311
**Cohort 3 (3)**	VALENCIA	*INCLIVAs Research Institute. Hospital Clinico Universitario de Valencia*	0	285	723	1008
			**2207**	**2420**	**1304**	**5931**

Supplementary table1: Final number and nature of the samples used in the study. Different cohorts are based on Country and Institution source. Originally, the number was larger; however, all samples with low quality of DNA, mislabeling or possible cross contamination were eliminated from the study.

	Basal	Her2	LumA	LumB	NORM_BREAST
**GSE10810**	6	4	8	12	27
**GSE12276**	57	31	56	58	0
**GSE12763/GSE12790**	5	5	6	14	0
**GSE16446**	70	15	22	4	0
**GSE18728**	22	3	18	10	0
**GSE18864**	36	8	21	7	0
**GSE19615**	28	16	41	30	0
**GSE20685**	43	57	118	93	0
**GSE20711**	23	17	17	31	2
**GSE2109**	73	41	123	101	0
**GSE22849**	3	3	2	5	4
**GSE23177**	7	7	40	59	0
**GSE23593**	4	2	7	2	0
**GSE26639**	43	37	75	69	0
**GSE28821**	18	14	20	30	0
**GSE29044**	6	9	9	14	36
**GSE29431**	4	8	13	20	12
**GSE31448**	104	43	98	98	0
**GSE32646**	19	28	23	36	0
**GSE36771/GSE36774**	20	14	34	35	0
**GSE42568**	16	15	18	44	16
**GSE43358**	16	5	22	14	0
**GSE45827**	41	28	25	36	11
**GSE48390/GSE48391**	8	10	20	32	0
**GSE50567**	22	3	5	5	6

**GSE52322**	15	2	10	8	0
**GSE54002**	59	71	88	199	16
**GSE54219**	54	5	55	39	0
**GSE5460**	32	19	46	29	0
**GSE5764**	0	1	2	3	7
**GSE58812**	74	10	11	8	0
**GSE65194/GSE65216**	56	36	26	35	11
**GSE6532**	8	6	30	39	0
**GSE66305/GSE66399**	9	29	16	30	0
**GSE76124/GSE76275**	134	10	24	19	0
**GSE76274/GSE76275**	1	6	33	27	0
**GSE7904**	20	7	3	12	19
**GSE88770**	14	7	39	40	0
**GSE9195**	5	7	29	32	0
**Total**	1175	639	1253	1379	167

Supplementary table 2: Studies included in the D1 dataset. Columns indicate the number of healthy breast cancer samples and the number of each breast cancer subtype samples present in each study.

NAME	SIZE	ES	NES	NOM p-val	FDR q-val	FWER p-val	PER3 Pos Neg
KEGG_VALINE_LEUCINE_AND_ISOLEUCINE_DEGRADATION	**42**	**0.69**	**2.59**	**0.00E+00**	**0.00E+00**	**0.00E+00**	**Pos**
REACTOME_PYRUVATE_METABOLISM_AND_CITRIC_ACID_TCA_CYCLE	**37**	**0.70**	**2.59**	**0.00E+00**	**0.00E+00**	**0.00E+00**	**Pos**
KEGG_CITRATE_CYCLE_TCA_CYCLE	**29**	**0.72**	**2.48**	**0.00E+00**	**0.00E+00**	**0.00E+00**	**Pos**
REACTOME_TCA_CYCLE_AND_RESPIRATORY_ELECTRON_TRANSPORT	**90**	**0.56**	**2.42**	**0.00E+00**	**0.00E+00**	**0.00E+00**	**Pos**
KEGG_PROPANOATE_METABOLISM	**29**	**0.69**	**2.41**	**0.00E+00**	**0.00E+00**	**0.00E+00**	**Pos**
PID_TCPTP_PATHWAY	**42**	**0.62**	**2.34**	**0.00E+00**	**1.78E-05**	**1.00E-04**	**Pos**
REACTOME_CITRIC_ACID_CYCLE_TCA_CYCLE	**18**	**0.75**	**2.33**	**0.00E+00**	**3.02E-05**	**2.00E-04**	**Pos**
KEGG_INSULIN_SIGNALING_PATHWAY	**132**	**0.50**	**2.30**	**0.00E+00**	**3.97E-05**	**3.00E-04**	**Pos**
BIOCARTA_TPO_PATHWAY	**22**	**0.70**	**2.25**	**0.00E+00**	**9.51E-05**	**8.00E-04**	**Pos**
BIOCARTA_GLEEVEC_PATHWAY	**22**	**0.68**	**2.21**	**0.00E+00**	**2.04E-04**	**1.90E-03**	**Pos**
BIOCARTA_PDGF_PATHWAY	**30**	**0.62**	**2.19**	**0.00E+00**	**4.92E-04**	**5.00E-03**	**Pos**
KEGG_PYRUVATE_METABOLISM	**37**	**0.59**	**2.18**	**0.00E+00**	**5.13E-04**	**5.70E-03**	**Pos**
BIOCARTA_EGF_PATHWAY	**29**	**0.62**	**2.16**	**0.00E+00**	**6.91E-04**	**8.30E-03**	**Pos**
SA_PTEN_PATHWAY	**16**	**0.72**	**2.15**	**1.46E-04**	**7.58E-04**	**9.80E-03**	**Pos**
REACTOME_FATTY_ACID_TRIACYLGLYCEROL_AND_KETONE_BODY_METABOLISM	**156**	**0.46**	**2.15**	**0.00E+00**	**7.22E-04**	**1.00E-02**	**Pos**
KEGG_FATTY_ACID_METABOLISM	**35**	**0.58**	**2.12**	**0.00E+00**	**1.20E-03**	**1.75E-02**	**Pos**
REACTOME_PEROXISOMAL_LIPID_METABOLISM	**20**	**0.67**	**2.11**	**0.00E+00**	**1.45E-03**	**2.25E-02**	**Pos**
REACTOME_BRANCHED_CHAIN_AMINO_ACID_CATABOLISM	**16**	**0.71**	**2.10**	**2.97E-04**	**1.46E-03**	**2.40E-02**	**Pos**
PID_IL2_1PATHWAY	**52**	**0.53**	**2.10**	**0.00E+00**	**1.55E-03**	**2.68E-02**	**Pos**
BIOCARTA_INSULIN_PATHWAY	**20**	**0.66**	**2.08**	**0.00E+00**	**1.81E-03**	**3.27E-02**	**Pos**
PID_IL6_7_PATHWAY	**45**	**0.55**	**2.08**	**0.00E+00**	**1.83E-03**	**3.47E-02**	**Pos**
PID_ERBB2_ERBB3_PATHWAY	**43**	**0.55**	**2.08**	**0.00E+00**	**1.79E-03**	**3.56E-02**	**Pos**
KEGG_PPAR_SIGNALING_PATHWAY	**64**	**0.51**	**2.07**	**2.46E-04**	**1.82E-03**	**3.80E-02**	**Pos**
PID_FAK_PATHWAY	**57**	**0.52**	**2.07**	**0.00E+00**	**1.79E-03**	**3.89E-02**	**Pos**
PID_ERBB1_INTERNALIZATION_PATHWAY	**38**	**0.56**	**2.07**	**0.00E+00**	**1.80E-03**	**4.09E-02**	**Pos**
PID_PDGFRA_PATHWAY	**20**	**0.65**	**2.07**	**0.00E+00**	**1.88E-03**	**4.42E-02**	**Pos**
BIOCARTA_PTEN_PATHWAY	**18**	**0.67**	**2.06**	**0.00E+00**	**1.84E-03**	**4.49E-02**	**Pos**
REACTOME_RESPIRATORY_ELECTRON_TRANSPORT	**54**	**0.52**	**2.06**	**0.00E+00**	**1.90E-03**	**4.81E-02**	**Pos**
BIOCARTA_MET_PATHWAY	**36**	**0.56**	**2.04**	**1.33E-04**	**2.41E-03**	**6.25E-02**	**Pos**
REACTOME_AUTODEGRADATION_OF_CDH1_BY_CDH1_APC_C	**52**	**0.52**	**2.04**	**0.00E+00**	**2.52E-03**	**6.73E-02**	**Pos**
BIOCARTA_PPARA_PATHWAY	**53**	**0.51**	**2.04**	**0.00E+00**	**2.53E-03**	**6.97E-02**	**Pos**
PID_INSULIN_PATHWAY	**44**	**0.53**	**2.03**	**0.00E+00**	**2.61E-03**	**7.38E-02**	**Pos**
REACTOME_RESPIRATORY_ELECTRON_TRANSPORT_ATP_SYNTHESIS_BY_CHEMIOSMOTIC_COUPLING_AND_HEAT_PRODUCTION_BY_UNCOUPLING_PROTEINS_	**57**	**0.51**	**2.03**	**0.00E+00**	**2.73E-03**	**7.98E-02**	**Pos**
REACTOME_ANTIGEN_PROCESSING_UBIQUITINATION_PROTEASOME_DEGRADATION	**179**	**0.43**	**2.02**	**0.00E+00**	**2.97E-03**	**8.94E-02**	**Pos**
REACTOME_CLASS_I_MHC_MEDIATED_ANTIGEN_PROCESSING_PRESENTATION	**210**	**0.42**	**2.02**	**0.00E+00**	**3.02E-03**	**9.37E-02**	**Pos**
BIOCARTA_IGF1_PATHWAY	**19**	**0.64**	**2.00**	**1.45E-04**	**3.67E-03**	**1.15E-01**	**Pos**
PID_ERBB1_RECEPTOR_PROXIMAL_PATHWAY	**34**	**0.56**	**2.00**	**0.00E+00**	**3.66E-03**	**1.18E-01**	**Pos**
BIOCARTA_IGF1MTOR_PATHWAY	**19**	**0.64**	**2.00**	**2.89E-04**	**3.74E-03**	**1.23E-01**	**Pos**
REACTOME_TRANSCRIPTIONAL_REGULATION_OF_WHITE_ADIPOCYTE_DIFFERENTIATION	**67**	**0.48**	**2.00**	**0.00E+00**	**3.71E-03**	**1.25E-01**	**Pos**
PID_VEGFR1_2_PATHWAY	**66**	**0.49**	**1.99**	**1.23E-04**	**3.94E-03**	**1.36E-01**	**Pos**

KEGG_UBIQUITIN_MEDIATED_PROTEOLYSIS	115	0.44	1.99	0.00E+00	4.05E-03	1.43E-01	Pos
REACTOME_DOWNSTREAM_SIGNAL_TRANSDUCTION	85	0.46	1.98	0.00E+00	4.31E-03	1.55E-01	Pos
REACTOME_SIGNALING_BY_EGFR_IN_CANCER	99	0.45	1.97	0.00E+00	4.60E-03	1.68E-01	Pos
PID_MTOR_4PATHWAY	63	0.48	1.97	1.23E-04	4.56E-03	1.70E-01	Pos
REACTOME_PYRUVATE_METABOLISM	16	0.66	1.97	4.46E-04	4.56E-03	1.74E-01	Pos
REACTOME_GPVI_MEDIATED_ACTIVATION_CASCADE	30	0.56	1.97	1.36E-04	4.58E-03	1.77E-01	Pos
REACTOME_SIGNALING_BY_WNT	56	0.50	1.97	0.00E+00	4.49E-03	1.78E-01	Pos
PID_AR_TF_PATHWAY	48	0.51	1.97	0.00E+00	4.40E-03	1.78E-01	Pos
BIOCARTA_PROTEASOME_PATHWAY	27	0.58	1.97	0.00E+00	4.32E-03	1.78E-01	Pos
REACTOME_PI3K_AKT_ACTIVATION	35	0.54	1.97	2.65E-04	4.41E-03	1.85E-01	Pos
BIOCARTA_IL6_PATHWAY	20	0.62	1.96	7.17E-04	4.35E-03	1.86E-01	Pos
PID_PDGFRB_PATHWAY	124	0.43	1.96	0.00E+00	4.28E-03	1.87E-01	Pos
PID_GMCSF_PATHWAY	33	0.55	1.96	4.02E-04	4.48E-03	1.98E-01	Pos
REACTOME_YAP1_AND_WWTR1_TAZ_STIMULATED_GENE_EXPRESSION	22	0.60	1.95	2.84E-04	5.04E-03	2.24E-01	Pos
PID_CDC42_REG_PATHWAY	27	0.57	1.95	5.52E-04	4.97E-03	2.25E-01	Pos
REACTOME_NGF_SIGNALLING_VIA_TRKA_FROM_THE_PLASMA_MEMBRANE	126	0.43	1.95	0.00E+00	5.04E-03	2.31E-01	Pos
REACTOME_SIGNALING_BY_BMP	22	0.60	1.95	7.09E-04	5.11E-03	2.38E-01	Pos
PID_S1P_S1P3_PATHWAY	29	0.56	1.94	5.44E-04	5.11E-03	2.41E-01	Pos
KEGG_BUTANOATE_METABOLISM	31	0.55	1.94	1.33E-04	5.24E-03	2.51E-01	Pos
REACTOME_REGULATION_OF_ORNITHINE_DECARBOXYLASE_ODC	45	0.51	1.94	6.42E-04	5.23E-03	2.54E-01	Pos
PID_SHP2_PATHWAY	53	0.49	1.94	2.52E-04	5.40E-03	2.63E-01	Pos
SIG_IL4RECEPTOR_IN_B_LYPHOCYTES	26	0.57	1.93	6.91E-04	5.57E-03	2.75E-01	Pos
PID_THROMBIN_PAR1_PATHWAY	41	0.51	1.93	1.31E-04	5.62E-03	2.81E-01	Pos
PID_ANGIOPOIETIN_RECEPTOR_PATHWAY	49	0.49	1.93	0.00E+00	5.73E-03	2.90E-01	Pos
KEGG_COLORECTAL_CANCER	62	0.47	1.92	0.00E+00	5.76E-03	2.95E-01	Pos
REACTOME_EGFR_DOWNREGULATION	24	0.58	1.92	5.54E-04	5.74E-03	2.98E-01	Pos
REACTOME_REGULATION_OF_MRNA_STABILITY_BY_PROTEINS_THAT_BIND_AU_RICH_ELEMENTS	73	0.46	1.92	0.00E+00	6.15E-03	3.19E-01	Pos
REACTOME_GLUCOSE_METABOLISM	58	0.47	1.91	4.99E-04	7.03E-03	3.58E-01	Pos
REACTOME_CIRCADIAN_CLOCK	46	0.50	1.90	3.83E-04	7.54E-03	3.82E-01	Pos
REACTOME_DOWNSTREAM_SIGNALING_EVENTS_OF_B_CELL_RECEPTOR_BCR	86	0.44	1.90	1.18E-04	7.45E-03	3.83E-01	Pos
KEGG_PEROXISOME	72	0.45	1.90	0.00E+00	7.36E-03	3.84E-01	Pos
REACTOME_VIF_MEDIATED_DEGRADATION_OF_APOBEC3G	43	0.50	1.90	9.03E-04	7.75E-03	4.02E-01	Pos
REACTOME_SIGNALING_BY_ERBB4	82	0.44	1.90	0.00E+00	7.65E-03	4.03E-01	Pos
REACTOME_GOLGI_ASSOCIATED_VESICLE_BIOGENESIS	48	0.49	1.89	0.00E+00	7.91E-03	4.18E-01	Pos
REACTOME_PIP3_ACTIVATES_AKT_SIGNALING	26	0.56	1.89	8.30E-04	7.83E-03	4.19E-01	Pos
REACTOME_SIGNALING_BY_PDGF	111	0.42	1.89	0.00E+00	7.74E-03	4.20E-01	Pos
KEGG_SPLICEOSOME	99	0.43	1.89	0.00E+00	7.70E-03	4.23E-01	Pos
KEGG_ENDOMETRIAL_CANCER	51	0.48	1.88	1.27E-04	8.40E-03	4.54E-01	Pos
KEGG_ENDOCYTOSIS	154	0.40	1.88	0.00E+00	8.73E-03	4.71E-01	Pos
REACTOME_ASPARAGINE_N_LINKED_GLYCOSYLATION	72	0.45	1.88	1.21E-04	8.62E-03	4.71E-01	Pos
SIG_INSULIN_RECEPTOR_PATHWAY_IN_CARDIAC_MYOCYTES	48	0.48	1.88	3.83E-04	8.53E-03	4.72E-01	Pos

REACTOME_TRANS_GOLGI_NETWORK_VESICLE_BUDDING	55	0.47	1.88	5.01E-04	8.46E-03	4.73E-01	Pos
PID_IGF1_PATHWAY	28	0.55	1.88	8.19E-04	8.36E-03	4.73E-01	Pos
PID_MET_PATHWAY	76	0.44	1.87	2.40E-04	8.93E-03	4.98E-01	Pos
PID_RXR_VDR_PATHWAY	26	0.55	1.87	1.79E-03	8.89E-03	5.00E-01	Pos
PID_CXCR4_PATHWAY	97	0.43	1.87	1.16E-04	8.94E-03	5.05E-01	Pos
REACTOME_SIGNALING_BY_CONSTITUTIVELY_ACTIVE_EGFR	16	0.63	1.87	1.90E-03	8.83E-03	5.05E-01	Pos
PID_AVB3_OPN_PATHWAY	30	0.53	1.87	1.51E-03	9.01E-03	5.16E-01	Pos
KEGG_REGULATION_OF_AUTOPHAGY	21	0.58	1.87	1.55E-03	8.98E-03	5.19E-01	Pos
PID_KIT_PATHWAY	50	0.48	1.86	1.29E-04	9.07E-03	5.26E-01	Pos
REACTOME_GAB1_SIGNALOSOME	35	0.51	1.86	1.06E-03	9.44E-03	5.44E-01	Pos
REACTOME_MEMBRANE_TRAFFICKING	115	0.41	1.86	0.00E+00	9.64E-03	5.55E-01	Pos
PID_TGFBR_PATHWAY	52	0.47	1.85	1.26E-04	9.92E-03	5.70E-01	Pos
BIOCARTA_IL7_PATHWAY	17	0.61	1.85	1.32E-03	1.03E-02	5.87E-01	Pos
KEGG_RENAL_CELL_CARCINOMA	62	0.45	1.85	8.65E-04	1.05E-02	6.01E-01	Pos
PID_IL1_PATHWAY	32	0.52	1.85	1.36E-03	1.04E-02	6.01E-01	Pos
PID_PI3KCI_AKT_PATHWAY	34	0.51	1.84	1.06E-03	1.06E-02	6.10E-01	Pos
PID_NCADHERIN_PATHWAY	31	0.52	1.84	1.36E-03	1.10E-02	6.26E-01	Pos
BIOCARTA_GSK3_PATHWAY	27	0.54	1.84	2.33E-03	1.09E-02	6.29E-01	Pos
BIOCARTA_IL2_PATHWAY	19	0.59	1.84	2.29E-03	1.11E-02	6.38E-01	Pos
BIOCARTA_ECM_PATHWAY	23	0.56	1.84	2.37E-03	1.13E-02	6.50E-01	Pos
BIOCARTA_SPRY_PATHWAY	17	0.60	1.83	2.93E-03	1.14E-02	6.55E-01	Pos
BIOCARTA_MTOR_PATHWAY	21	0.57	1.83	1.70E-03	1.13E-02	6.55E-01	Pos
REACTOME_ANTIVIRAL_MECHANISM_BY_IFN_STIMULATED_GENES	56	0.46	1.83	3.76E-04	1.13E-02	6.61E-01	Pos
REACTOME_DESTABILIZATION_OF_MRNA_BY_AUF1_HNRNP_D0	44	0.48	1.83	1.29E-03	1.13E-02	6.62E-01	Pos
REACTOME_RNA_POL_II_TRANSCRIPTION_PRE_INITIATION_AND_PROMOTER_OPENING	36	0.50	1.83	1.21E-03	1.12E-02	6.64E-01	Pos
KEGG_ACUTE_MYELOID_LEUKEMIA	54	0.46	1.83	6.28E-04	1.16E-02	6.82E-01	Pos
PID_VEGFR1_PATHWAY	26	0.54	1.83	2.36E-03	1.16E-02	6.84E-01	Pos
BIOCARTA_TFF_PATHWAY	20	0.58	1.83	3.16E-03	1.15E-02	6.86E-01	Pos
PID_AR_PATHWAY	54	0.46	1.82	6.30E-04	1.20E-02	7.04E-01	Pos
PID_BMP_PATHWAY	40	0.49	1.82	1.46E-03	1.20E-02	7.07E-01	Pos
KEGG_MTOR_SIGNALING_PATHWAY	50	0.46	1.82	1.01E-03	1.20E-02	7.11E-01	Pos
ST_INTEGRIN_SIGNALING_PATHWAY	78	0.43	1.82	4.81E-04	1.21E-02	7.18E-01	Pos
REACTOME_SIGNALLING_BY_NGF	201	0.38	1.82	1.07E-04	1.20E-02	7.18E-01	Pos
REACTOME_PI_METABOLISM	44	0.48	1.82	1.83E-03	1.25E-02	7.37E-01	Pos
PID_ECADHERIN_KERATINOCYTE_PATHWAY	19	0.58	1.81	3.61E-03	1.26E-02	7.44E-01	Pos
PID_IFNG_PATHWAY	39	0.49	1.81	1.45E-03	1.27E-02	7.50E-01	Pos
BIOCARTA_PAR1_PATHWAY	36	0.49	1.81	1.33E-03	1.29E-02	7.58E-01	Pos
PID_IL3_PATHWAY	24	0.54	1.81	2.81E-03	1.28E-02	7.58E-01	Pos
BIOCARTA_RARRXR_PATHWAY	15	0.62	1.81	3.38E-03	1.29E-02	7.66E-01	Pos
PID_WNT_CANONICAL_PATHWAY	17	0.59	1.81	4.07E-03	1.32E-02	7.75E-01	Pos
BIOCARTA_EPO_PATHWAY	17	0.59	1.80	3.79E-03	1.37E-02	7.90E-01	Pos

PID_ECADHERIN_NASCENT_AJ_PATHWAY	**35**	**0.50**	**1.80**	**1.32E-03**	**1.37E-02**	**7.92E-01**	**Pos**
REACTOME_AUTODEGRADATION_OF_THE_E3_UBIQUITIN_LIGASE_COP1	**43**	**0.48**	**1.80**	**1.29E-03**	**1.37E-02**	**7.94E-01**	**Pos**
PID_RHOA_PATHWAY	**42**	**0.48**	**1.80**	**2.21E-03**	**1.36E-02**	**7.94E-01**	**Pos**
PID_CXCR3_PATHWAY	**41**	**0.48**	**1.80**	**1.70E-03**	**1.37E-02**	**8.02E-01**	**Pos**
BIOCARTA_AKT_PATHWAY	**22**	**0.55**	**1.79**	**3.71E-03**	**1.50E-02**	**8.31E-01**	**Pos**
PID_ERBB1_DOWNSTREAM_PATHWAY	**103**	**0.41**	**1.79**	**2.31E-04**	**1.49E-02**	**8.32E-01**	**Pos**
REACTOME_PROCESSING_OF_CAPPED_INTRON_CONTAINING_PRE_MRNA	**113**	**0.40**	**1.79**	**0.00E+00**	**1.49E-02**	**8.34E-01**	**Pos**
SIG_PIP3_SIGNALING_IN_CARDIAC_MYOCTES	**63**	**0.44**	**1.79**	**1.36E-03**	**1.48E-02**	**8.35E-01**	**Pos**
REACTOME_METABOLISM_OF_LIPIDS_AND_LIPOPROTEINS	**422**	**0.36**	**1.79**	**0.00E+00**	**1.53E-02**	**8.46E-01**	**Pos**
PID_MYC_REPRESS_PATHWAY	**57**	**0.44**	**1.79**	**1.00E-03**	**1.54E-02**	**8.49E-01**	**Pos**
REACTOME_TRIGLYCERIDE_BIOSYNTHESIS	**35**	**0.49**	**1.78**	**1.99E-03**	**1.54E-02**	**8.52E-01**	**Pos**
REACTOME_RNA_POL_II_PRE_TRANSCRIPTION_EVENTS	**45**	**0.47**	**1.78**	**2.05E-03**	**1.56E-02**	**8.56E-01**	**Pos**
PID_ER_NONGENOMIC_PATHWAY	**40**	**0.48**	**1.78**	**2.10E-03**	**1.58E-02**	**8.63E-01**	**Pos**
REACTOME_ACTIVATION_OF_NF_KAPPAB_IN_B_CELLS	**57**	**0.44**	**1.78**	**1.62E-03**	**1.57E-02**	**8.63E-01**	**Pos**
PID_PTP1B_PATHWAY	**50**	**0.45**	**1.78**	**1.40E-03**	**1.60E-02**	**8.70E-01**	**Pos**
BIOCARTA_NGF_PATHWAY	**16**	**0.60**	**1.78**	**5.66E-03**	**1.62E-02**	**8.76E-01**	**Pos**
KEGG_PARKINSONS_DISEASE	**85**	**0.41**	**1.77**	**3.53E-04**	**1.66E-02**	**8.83E-01**	**Pos**
PID_HEDGEHOG_GLI_PATHWAY	**45**	**0.46**	**1.77**	**2.19E-03**	**1.70E-02**	**8.92E-01**	**Pos**
REACTOME_SIGNALING_BY_THE_B_CELL_RECEPTOR_BCR	**113**	**0.40**	**1.77**	**3.42E-04**	**1.72E-02**	**8.97E-01**	**Pos**
BIOCARTA_HCMV_PATHWAY	**16**	**0.59**	**1.77**	**6.30E-03**	**1.73E-02**	**8.98E-01**	**Pos**
KEGG_NEUROTROPHIN_SIGNALING_PATHWAY	**120**	**0.39**	**1.76**	**0.00E+00**	**1.77E-02**	**9.05E-01**	**Pos**
REACTOME_REGULATION_OF_SIGNALING_BY_CBL	**17**	**0.58**	**1.76**	**5.46E-03**	**1.76E-02**	**9.05E-01**	**Pos**
PID_ENDOTHELIN_PATHWAY	**61**	**0.43**	**1.76**	**6.16E-04**	**1.79E-02**	**9.11E-01**	**Pos**
PID_EPHB_FWD_PATHWAY	**39**	**0.47**	**1.76**	**2.22E-03**	**1.79E-02**	**9.14E-01**	**Pos**
BIOCARTA_HDAC_PATHWAY	**27**	**0.52**	**1.76**	**3.59E-03**	**1.79E-02**	**9.14E-01**	**Pos**
REACTOME_SIGNALING_BY_FGFR1_FUSION_MUTANTS	**15**	**0.60**	**1.76**	**6.75E-03**	**1.78E-02**	**9.14E-01**	**Pos**
KEGG_PROTEASOME	**40**	**0.47**	**1.76**	**2.88E-03**	**1.77E-02**	**9.14E-01**	**Pos**
PID_LYSOPHOSPHOLIPID_PATHWAY	**64**	**0.43**	**1.76**	**1.35E-03**	**1.80E-02**	**9.21E-01**	**Pos**
BIOCARTA_EIF4_PATHWAY	**22**	**0.54**	**1.76**	**6.87E-03**	**1.80E-02**	**9.21E-01**	**Pos**
BIOCARTA_INTEGRIN_PATHWAY	**37**	**0.48**	**1.76**	**2.95E-03**	**1.79E-02**	**9.22E-01**	**Pos**
REACTOME_ACTIVATION_OF_CHAPERONE_GENES_BY_XBP1S	**34**	**0.49**	**1.76**	**4.41E-03**	**1.81E-02**	**9.25E-01**	**Pos**
REACTOME_RORA_ACTIVATES_CIRCADIAN_EXPRESSION	**21**	**0.55**	**1.76**	**6.70E-03**	**1.80E-02**	**9.26E-01**	**Pos**
REACTOME_BMAL1_CLOCK_NPAS2_ACTIVATES_CIRCADIAN_EXPRESSION	**31**	**0.50**	**1.75**	**3.54E-03**	**1.82E-02**	**9.29E-01**	**Pos**
KEGG_LYSINE_DEGRADATION	**36**	**0.48**	**1.75**	**3.57E-03**	**1.84E-02**	**9.32E-01**	**Pos**
REACTOME_PPARA_ACTIVATES_GENE_EXPRESSION	**97**	**0.40**	**1.75**	**2.31E-04**	**1.83E-02**	**9.33E-01**	**Pos**
REACTOME_MRNA_PROCESSING	**130**	**0.38**	**1.75**	**1.12E-04**	**1.85E-02**	**9.37E-01**	**Pos**
REACTOME_PHOSPHOLIPID_METABOLISM	**168**	**0.37**	**1.75**	**1.08E-04**	**1.87E-02**	**9.40E-01**	**Pos**
ST_ERK1_ERK2_MAPK_PATHWAY	**30**	**0.50**	**1.75**	**3.70E-03**	**1.88E-02**	**9.42E-01**	**Pos**
REACTOME_SCF_BETA_TRCP_MEDIATED_DEGRADATION_OF_EMI1	**45**	**0.46**	**1.75**	**2.05E-03**	**1.90E-02**	**9.44E-01**	**Pos**
KEGG_PROXIMAL_TUBULE_BICARBONATE_RECLAMATION	**22**	**0.54**	**1.75**	**7.84E-03**	**1.91E-02**	**9.46E-01**	**Pos**
PID_S1P_S1P2_PATHWAY	**24**	**0.52**	**1.75**	**6.96E-03**	**1.90E-02**	**9.46E-01**	**Pos**

PID_TELOMERASE_PATHWAY	63	0.43	1.74	1.11E-03	1.90E-02	9.47E-01	Pos
ST_GA13_PATHWAY	34	0.48	1.74	3.88E-03	1.91E-02	9.48E-01	Pos
KEGG_BIOSYNTHESIS_OF_UNSATURATED_FATTY_ACIDS	16	0.58	1.74	5.75E-03	1.91E-02	9.49E-01	Pos
ST_ADRENERGIC	33	0.49	1.74	4.17E-03	1.90E-02	9.49E-01	Pos
BIOCARTA_MAPK_PATHWAY	84	0.41	1.74	4.72E-04	1.91E-02	9.50E-01	Pos
KEGG_N_GLYCAN_BIOSYNTHESIS	40	0.47	1.74	3.86E-03	1.97E-02	9.55E-01	Pos
PID_IL8_CXCR1_PATHWAY	24	0.52	1.73	7.30E-03	2.07E-02	9.62E-01	Pos
PID_ARF6_TRAFFICKING_PATHWAY	47	0.45	1.73	2.18E-03	2.07E-02	9.63E-01	Pos
REACTOME_SIGNALING_BY_ERBB2	91	0.40	1.73	8.20E-04	2.08E-02	9.64E-01	Pos
REACTOME_CYCLIN_E_ASSOCIATED_EVENTS_DURING_G1_S_TRANSITION_	58	0.43	1.73	1.74E-03	2.11E-02	9.66E-01	Pos
REACTOME_CDK_MEDIATED_PHOSPHORYLATION_AND_REMOVAL_OF_CDC6	43	0.46	1.73	3.40E-03	2.16E-02	9.70E-01	Pos
BIOCARTA_CARDIACEGF_PATHWAY	17	0.57	1.72	7.64E-03	2.22E-02	9.73E-01	Pos
REACTOME_RNA_POL_II_TRANSCRIPTION	76	0.41	1.72	1.44E-03	2.22E-02	9.74E-01	Pos
PID_RET_PATHWAY	37	0.47	1.72	5.56E-03	2.21E-02	9.74E-01	Pos
PID_ILK_PATHWAY	43	0.45	1.72	4.68E-03	2.31E-02	9.78E-01	Pos
KEGG_NOTCH_SIGNALING_PATHWAY	47	0.44	1.71	3.10E-03	2.46E-02	9.84E-01	Pos
SA_TRKA_RECEPTOR	16	0.57	1.71	1.05E-02	2.45E-02	9.84E-01	Pos
BIOCARTA_BCELLSURVIVAL_PATHWAY	15	0.58	1.71	1.05E-02	2.46E-02	9.85E-01	Pos
ST_JNK_MAPK_PATHWAY	38	0.46	1.71	3.82E-03	2.53E-02	9.87E-01	Pos
KEGG_FOCAL_ADHESION	184	0.36	1.71	0.00E+00	2.54E-02	9.88E-01	Pos
REACTOME_PI3K_EVENTS_IN_ERBB2_SIGNALING	41	0.45	1.71	4.95E-03	2.54E-02	9.88E-01	Pos
BIOCARTA_FCER1_PATHWAY	35	0.47	1.70	5.16E-03	2.54E-02	9.88E-01	Pos
REACTOME_TRANSPORT_OF_MATURE_TRANSCRIPT_TO_CYTOPLASM	39	0.46	1.70	5.00E-03	2.55E-02	9.89E-01	Pos
REACTOME_HIV_INFECTION	167	0.36	1.70	1.09E-04	2.61E-02	9.91E-01	Pos
REACTOME_ADAPTIVE_IMMUNE_SYSTEM	439	0.34	1.70	0.00E+00	2.66E-02	9.92E-01	Pos
REACTOME_GLUCONEOGENESIS	28	0.49	1.70	7.61E-03	2.66E-02	9.92E-01	Pos
PID_PI3K_PLC_TRK_PATHWAY	34	0.47	1.70	6.03E-03	2.66E-02	9.92E-01	Pos
KEGG_AMINO_SUGAR_AND_NUCLEOTIDE_SUGAR_METABOLISM	39	0.46	1.70	5.63E-03	2.65E-02	9.92E-01	Pos
REACTOME_SIGNALING_BY_NOTCH	91	0.39	1.70	3.55E-04	2.64E-02	9.92E-01	Pos
BIOCARTA_RAS_PATHWAY	22	0.52	1.70	1.03E-02	2.66E-02	9.93E-01	Pos
BIOCARTA_IGF1R_PATHWAY	21	0.53	1.70	9.12E-03	2.65E-02	9.93E-01	Pos
BIOCARTA_CHREBP2_PATHWAY	39	0.45	1.69	6.00E-03	2.66E-02	9.93E-01	Pos
REACTOME_CD28_CO_STIMULATION	31	0.48	1.69	6.92E-03	2.66E-02	9.94E-01	Pos
REACTOME_LATE_PHASE_OF_HIV_LIFE_CYCLE	83	0.40	1.69	2.38E-03	2.65E-02	9.94E-01	Pos
KEGG_HUNTINGTONS_DISEASE	138	0.37	1.69	3.33E-04	2.64E-02	9.94E-01	Pos
REACTOME_SIGNALING_BY_SCF_KIT	71	0.40	1.69	2.07E-03	2.65E-02	9.94E-01	Pos
REACTOME_FORMATION_OF_INCISION_COMPLEX_IN_GG_NER	18	0.55	1.69	9.89E-03	2.69E-02	9.94E-01	Pos
BIOCARTA_CDC42RAC_PATHWAY	16	0.56	1.69	1.51E-02	2.75E-02	9.95E-01	Pos
BIOCARTA_VEGF_PATHWAY	25	0.50	1.69	9.59E-03	2.79E-02	9.95E-01	Pos
REACTOME_DOWNREGULATION_OF_TGF_BETA_RECEPTOR_SIGNALING	21	0.53	1.69	1.07E-02	2.78E-02	9.95E-01	Pos
REACTOME_NFKB_AND_MAP_KINASES_ACTIVATION_MEDIATED_BY_TLR4_SIGNALING_REPERTOIRE	66	0.41	1.69	3.45E-03	2.77E-02	9.95E-01	Pos

REACTOME_SCFSKP2_MEDIATED_DEGRADATION_OF_P27_P21	49	0.43	1.69	5.72E-03	2.77E-02	9.95E-01	Pos
REACTOME_G_ALPHA1213_SIGNALLING_EVENTS	71	0.40	1.68	2.79E-03	2.78E-02	9.96E-01	Pos
PID_SMAD2_3PATHWAY	17	0.55	1.68	1.36E-02	2.89E-02	9.96E-01	Pos
KEGG_NUCLEOTIDE_EXCISION_REPAIR	40	0.45	1.68	6.15E-03	2.89E-02	9.96E-01	Pos
REACTOME_NUCLEOTIDE_EXCISION_REPAIR	44	0.44	1.68	6.51E-03	2.91E-02	9.97E-01	Pos
REACTOME_ACTIVATED_AMPK_STIMULATES_FATTY_ACID_OXIDATION_IN_MUSCLE	17	0.55	1.68	1.30E-02	2.91E-02	9.97E-01	Pos
REACTOME_GENERIC_TRANSCRIPTION_PATHWAY	191	0.35	1.68	0.00E+00	2.90E-02	9.97E-01	Pos
REACTOME_RNA_POL_I_TRANSCRIPTION_INITIATION	21	0.52	1.68	1.21E-02	2.93E-02	9.97E-01	Pos
ST_INTERLEUKIN_4_PATHWAY	24	0.50	1.68	9.95E-03	2.92E-02	9.97E-01	Pos
REACTOME_ER_PHAGOSOME_PATHWAY	50	0.43	1.67	6.57E-03	2.94E-02	9.97E-01	Pos
KEGG_TGF_BETA_SIGNALING_PATHWAY	79	0.40	1.67	2.77E-03	2.94E-02	9.97E-01	Pos
REACTOME_CROSS_PRESENTATION_OF_SOLUBLE_EXOGENOUS_ANTIGENS_ENDOSOMES	43	0.44	1.67	8.40E-03	2.95E-02	9.97E-01	Pos
REACTOME_ANTIGEN_PRESENTATION_FOLDING_ASSEMBLY_AND_PEPTIDE_LOADING_OF_CLASS_I_MHC	15	0.57	1.67	1.44E-02	2.94E-02	9.97E-01	Pos
REACTOME_SIGNALING_BY_FGFR_IN_DISEASE	113	0.37	1.67	1.60E-03	2.94E-02	9.97E-01	Pos
REACTOME_POST_TRANSLATIONAL_MODIFICATION_SYNTHESIS_OF_GPI_ANCHORED_PROTEINS	26	0.49	1.67	9.59E-03	2.95E-02	9.98E-01	Pos
KEGG_ADIPOCYTOKINE_SIGNALING_PATHWAY	66	0.41	1.67	3.64E-03	2.97E-02	9.98E-01	Pos
BIOCARTA_TOLL_PATHWAY	35	0.46	1.67	8.28E-03	3.00E-02	9.98E-01	Pos
BIOCARTA_KERATINOCYTE_PATHWAY	43	0.44	1.67	6.70E-03	3.04E-02	9.98E-01	Pos
REACTOME_P53_DEPENDENT_G1_DNA_DAMAGE_RESPONSE	49	0.43	1.67	5.20E-03	3.03E-02	9.98E-01	Pos
PID_FGF_PATHWAY	52	0.42	1.67	4.78E-03	3.06E-02	9.98E-01	Pos
BIOCARTA_BIOPEPTIDES_PATHWAY	39	0.45	1.66	5.52E-03	3.06E-02	9.98E-01	Pos
PID_NFAT_3PATHWAY	48	0.43	1.66	6.08E-03	3.05E-02	9.98E-01	Pos
KEGG_LYSOSOME	110	0.37	1.66	1.72E-03	3.06E-02	9.98E-01	Pos
PID_NECTIN_PATHWAY	26	0.49	1.66	1.34E-02	3.10E-02	9.99E-01	Pos
PID_TRKR_PATHWAY	59	0.41	1.66	3.87E-03	3.09E-02	9.99E-01	Pos
BIOCARTA_ALK_PATHWAY	35	0.46	1.66	9.76E-03	3.13E-02	9.99E-01	Pos
REACTOME_PKB_MEDIATED_EVENTS	27	0.48	1.66	1.26E-02	3.19E-02	9.99E-01	Pos
KEGG_INOSITOL_PHOSPHATE_METABOLISM	54	0.42	1.66	6.39E-03	3.19E-02	9.99E-01	Pos
KEGG_FC_GAMMA_R_MEDIATED_PHAGOCYTOSIS	83	0.39	1.66	2.50E-03	3.19E-02	9.99E-01	Pos
REACTOME_CYTOKINE_SIGNALING_IN_IMMUNE_SYSTEM	213	0.35	1.65	1.06E-04	3.27E-02	9.99E-01	Pos
REACTOME_ANTIGEN_PROCESSING_CROSS_PRESENTATION	63	0.41	1.65	4.43E-03	3.26E-02	9.99E-01	Pos
BIOCARTA_TCR_PATHWAY	42	0.44	1.65	8.26E-03	3.25E-02	9.99E-01	Pos
KEGG_PANCREATIC_CANCER	68	0.40	1.65	5.14E-03	3.27E-02	9.99E-01	Pos
REACTOME_MAPK_TARGETS_NUCLEAR_EVENTS_MEDIATED_BY_MAP_KINASES	30	0.47	1.65	1.33E-02	3.38E-02	1.00E+00	Pos
PID_PS1_PATHWAY	43	0.43	1.65	1.06E-02	3.39E-02	1.00E+00	Pos
REACTOME_APOPTOTIC_CLEAVAGE_OF_CELLULAR_PROTEINS	34	0.46	1.65	1.00E-02	3.42E-02	1.00E+00	Pos
REACTOME_TGF_BETA_RECEPTOR_SIGNALING_ACTIVATES_SMADS	23	0.50	1.65	1.41E-02	3.41E-02	1.00E+00	Pos
REACTOME_RIG_I_MDA5_MEDIATED_INDUCTION_OF_IFN_ALPHA_BETA_PATHWAYS	53	0.42	1.64	7.11E-03	3.42E-02	1.00E+00	Pos
PID_SMAD2_3NUCLEAR_PATHWAY	76	0.39	1.64	3.11E-03	3.48E-02	1.00E+00	Pos
PID_HNF3A_PATHWAY	34	0.45	1.64	1.05E-02	3.48E-02	1.00E+00	Pos
KEGG_GLYOXYLATE_AND_DICARBOXYLATE_METABOLISM	16	0.55	1.64	1.66E-02	3.49E-02	1.00E+00	Pos

REACTOME_TRANSPORT_OF_RIBONUCLEOPROTEINS_INTO_THE_HOST_NUCLEUS	**24**	**0.49**	**1.64**	**1.64E-02**	**3.52E-02**	**1.00E+00**	**Pos**
BIOCARTA_NTHI_PATHWAY	**22**	**0.50**	**1.64**	**1.51E-02**	**3.57E-02**	**1.00E+00**	**Pos**
KEGG_CHRONIC_MYELOID_LEUKEMIA	**69**	**0.39**	**1.64**	**4.99E-03**	**3.57E-02**	**1.00E+00**	**Pos**
BIOCARTA_GH_PATHWAY	**27**	**0.48**	**1.64**	**1.71E-02**	**3.59E-02**	**1.00E+00**	**Pos**
REACTOME_TRAF6_MEDIATED_INDUCTION_OF_NFKB_AND_MAP_KINASES_UPON_TLR7_8_OR_9_ACTIVATION	**71**	**0.39**	**1.63**	**5.21E-03**	**3.64E-02**	**1.00E+00**	**Pos**
KEGG_GLUTATHIONE_METABOLISM	**39**	**0.44**	**1.63**	**1.13E-02**	**3.63E-02**	**1.00E+00**	**Pos**
PID_INSULIN_GLUCOSE_PATHWAY	**26**	**0.48**	**1.63**	**1.59E-02**	**3.71E-02**	**1.00E+00**	**Pos**
REACTOME_MYD88_MAL_CASCADE_INITIATED_ON_PLASMA_MEMBRANE	**77**	**0.39**	**1.63**	**3.72E-03**	**3.70E-02**	**1.00E+00**	**Pos**
REACTOME_MRNA_SPLICING	**87**	**0.38**	**1.63**	**3.77E-03**	**3.69E-02**	**1.00E+00**	**Pos**
REACTOME_FORMATION_OF_TRANSCRIPTION_COUPLED_NER_TC_NER_REPAIR_COMPLEX	**25**	**0.49**	**1.63**	**1.92E-02**	**3.73E-02**	**1.00E+00**	**Pos**
BIOCARTA_RELA_PATHWAY	**16**	**0.55**	**1.63**	**1.70E-02**	**3.79E-02**	**1.00E+00**	**Pos**
REACTOME_REGULATION_OF_APOPTOSIS	**53**	**0.41**	**1.63**	**7.26E-03**	**3.79E-02**	**1.00E+00**	**Pos**
REACTOME_CD28_DEPENDENT_PI3K_AKT_SIGNALING	**21**	**0.51**	**1.63**	**1.96E-02**	**3.82E-02**	**1.00E+00**	**Pos**
REACTOME_CIRCADIAN_REPRESSION_OF_EXPRESSION_BY_REV_ERBA	**20**	**0.51**	**1.62**	**2.00E-02**	**3.86E-02**	**1.00E+00**	**Pos**
REACTOME_UNFOLDED_PROTEIN_RESPONSE	**64**	**0.40**	**1.62**	**7.67E-03**	**3.85E-02**	**1.00E+00**	**Pos**
REACTOME_PRE_NOTCH_EXPRESSION_AND_PROCESSING	**36**	**0.44**	**1.62**	**1.41E-02**	**3.95E-02**	**1.00E+00**	**Pos**
REACTOME_METABOLISM_OF_VITAMINS_AND_COFACTORS	**47**	**0.42**	**1.62**	**8.83E-03**	**4.01E-02**	**1.00E+00**	**Pos**
REACTOME_SIGNALING_BY_TGF_BETA_RECEPTOR_COMPLEX	**58**	**0.40**	**1.62**	**9.09E-03**	**4.00E-02**	**1.00E+00**	**Pos**
KEGG_PROSTATE_CANCER	**86**	**0.38**	**1.62**	**4.94E-03**	**4.02E-02**	**1.00E+00**	**Pos**
PID_HDAC_CLASSI_PATHWAY	**62**	**0.40**	**1.62**	**7.32E-03**	**4.03E-02**	**1.00E+00**	**Pos**
PID_MYC_PATHWAY	**22**	**0.50**	**1.61**	**1.87E-02**	**4.06E-02**	**1.00E+00**	**Pos**
REACTOME_CDT1_ASSOCIATION_WITH_THE_CDC6_ORC_ORIGIN_COMPLEX	**45**	**0.42**	**1.61**	**1.26E-02**	**4.17E-02**	**1.00E+00**	**Pos**
REACTOME_PRE_NOTCH_TRANSCRIPTION_AND_TRANSLATION	**23**	**0.49**	**1.61**	**2.20E-02**	**4.20E-02**	**1.00E+00**	**Pos**
REACTOME_FORMATION_OF_RNA_POL_II_ELONGATION_COMPLEX_	**30**	**0.46**	**1.61**	**1.63E-02**	**4.20E-02**	**1.00E+00**	**Pos**
BIOCARTA_BAD_PATHWAY	**24**	**0.48**	**1.61**	**2.12E-02**	**4.30E-02**	**1.00E+00**	**Pos**
REACTOME_SYNTHESIS_OF_GLYCOSYLPHOSPHATIDYLINOSITOL_GPI	**17**	**0.53**	**1.61**	**2.37E-02**	**4.33E-02**	**1.00E+00**	**Pos**
REACTOME_ENDOSOMAL_SORTING_COMPLEX_REQUIRED_FOR_TRANSPORT_ESCRT	**22**	**0.49**	**1.60**	**2.30E-02**	**4.33E-02**	**1.00E+00**	**Pos**
BIOCARTA_PTDINS_PATHWAY	**22**	**0.49**	**1.60**	**2.03E-02**	**4.35E-02**	**1.00E+00**	**Pos**
REACTOME_PI3K_EVENTS_IN_ERBB4_SIGNALING	**35**	**0.44**	**1.60**	**1.54E-02**	**4.34E-02**	**1.00E+00**	**Pos**
PID_LYMPH_ANGIOGENESIS_PATHWAY	**24**	**0.48**	**1.60**	**1.82E-02**	**4.32E-02**	**1.00E+00**	**Pos**
PID_FCER1_PATHWAY	**55**	**0.40**	**1.60**	**8.68E-03**	**4.34E-02**	**1.00E+00**	**Pos**
REACTOME_INTERFERON_SIGNALING	**111**	**0.36**	**1.60**	**2.40E-03**	**4.36E-02**	**1.00E+00**	**Pos**
PID_AR_NONGENOMIC_PATHWAY	**30**	**0.46**	**1.60**	**1.83E-02**	**4.40E-02**	**1.00E+00**	**Pos**
PID_ALK1_PATHWAY	**24**	**0.48**	**1.60**	**1.80E-02**	**4.45E-02**	**1.00E+00**	**Pos**
REACTOME_IL_2_SIGNALING	**37**	**0.43**	**1.60**	**1.73E-02**	**4.45E-02**	**1.00E+00**	**Pos**
KEGG_GLYCOLYSIS_GLUCONEOGENESIS	**53**	**0.40**	**1.60**	**1.10E-02**	**4.44E-02**	**1.00E+00**	**Pos**
BIOCARTA_HER2_PATHWAY	**20**	**0.50**	**1.60**	**1.97E-02**	**4.43E-02**	**1.00E+00**	**Pos**
PID_ERBB4_PATHWAY	**38**	**0.43**	**1.60**	**1.74E-02**	**4.42E-02**	**1.00E+00**	**Pos**
ST_WNT_BETA_CATENIN_PATHWAY	**30**	**0.46**	**1.59**	**1.97E-02**	**4.55E-02**	**1.00E+00**	**Pos**
KEGG_BETA_ALANINE_METABOLISM	**20**	**0.50**	**1.59**	**2.50E-02**	**4.63E-02**	**1.00E+00**	**Pos**
REACTOME_APOPTOTIC_EXECUTION_PHASE	**47**	**0.41**	**1.59**	**1.27E-02**	**4.73E-02**	**1.00E+00**	**Pos**

PID_TXA2PATHWAY	53	0.40	1.59	9.90E-03	4.73E-02	1.00E+00	Pos
BIOCARTA_GPCR_PATHWAY	31	0.45	1.59	2.01E-02	4.73E-02	1.00E+00	Pos
PID_HIF1_TFPATHWAY	65	0.38	1.59	9.56E-03	4.77E-02	1.00E+00	Pos
REACTOME_P53_INDEPENDENT_G1_S_DNA_DAMAGE_CHECKPOINT	45	0.42	1.59	1.34E-02	4.79E-02	1.00E+00	Pos
PID_AVB3_INTEGRIN_PATHWAY	72	0.38	1.58	8.70E-03	4.80E-02	1.00E+00	Pos
REACTOME_SEMA4D_IN_SEMAPHORIN_SIGNALING	28	0.46	1.58	2.33E-02	4.90E-02	1.00E+00	Pos
REACTOME_SIGNALING_BY_NOTCH1	65	0.39	1.58	8.60E-03	4.92E-02	1.00E+00	Pos
REACTOME_ACTIVATED_TLR4_SIGNALLING	86	0.37	1.58	6.42E-03	4.93E-02	1.00E+00	Pos
PID_IL2_PI3K_PATHWAY	33	0.44	1.58	2.06E-02	4.94E-02	1.00E+00	Pos
REACTOME_APOPTOSIS	130	0.35	1.58	1.68E-03	4.92E-02	1.00E+00	Pos
ST_PHOSPHOINOSITIDE_3_KINASE_PATHWAY	35	0.44	1.58	1.81E-02	4.95E-02	1.00E+00	Pos
REACTOME_PEPTIDE_CHAIN_ELONGATION	60	-0.55	-2.78	0.00E+00	0.00E+00	0.00E+00	Neg
KEGG_RIBOSOME	63	-0.54	-2.72	0.00E+00	3.64E-05	1.00E-04	Neg
REACTOME_GPCR_LIGAND_BINDING	331	-0.40	-2.71	0.00E+00	2.42E-05	1.00E-04	Neg
REACTOME_PEPTIDE_LIGAND_BINDING_RECEPTORS	145	-0.45	-2.64	0.00E+00	1.82E-05	1.00E-04	Neg
KEGG_NEUROACTIVE_LIGAND_RECEPTOR_INTERACTION	244	-0.41	-2.63	0.00E+00	1.45E-05	1.00E-04	Neg
REACTOME_CLASS_A1_RHODOPSIN_LIKE_RECEPTORS	239	-0.41	-2.61	0.00E+00	1.21E-05	1.00E-04	Neg
KEGG_OLFACTORY_TRANSDUCTION	54	-0.53	-2.59	0.00E+00	3.10E-05	3.00E-04	Neg
PID_CONE_PATHWAY	20	-0.68	-2.52	0.00E+00	5.38E-05	6.00E-04	Neg
REACTOME_POTASSIUM_CHANNELS	93	-0.44	-2.40	0.00E+00	3.17E-04	4.00E-03	Neg
REACTOME_CHEMOKINE_RECEPTORS_BIND_CHEMOKINES	38	-0.52	-2.33	0.00E+00	6.49E-04	9.00E-03	Neg
REACTOME_OLFACTORY_SIGNALING_PATHWAY	31	-0.54	-2.27	0.00E+00	1.24E-03	1.86E-02	Neg
REACTOME_AMINE_COMPOUND_SLC_TRANSPORTERS	27	-0.55	-2.24	0.00E+00	1.54E-03	2.51E-02	Neg
REACTOME_3_UTR_MEDIATED_TRANSLATIONAL_REGULATION	74	-0.42	-2.23	0.00E+00	1.63E-03	2.86E-02	Neg
REACTOME_NA_CL_DEPENDENT_NEUROTRANSMITTER_TRANSPORTERS	17	-0.62	-2.19	3.22E-04	2.17E-03	4.09E-02	Neg
REACTOME_NONSENSE_MEDIATED_DECAY_ENHANCED_BY_THE_EXON_JUNCTION_COMPLEX	75	-0.41	-2.19	0.00E+00	2.25E-03	4.53E-02	Neg
REACTOME_LIGAND_GATED_ION_CHANNEL_TRANSPORT	20	-0.58	-2.17	6.54E-04	2.41E-03	5.19E-02	Neg
KEGG_MATURITY_ONSET_DIABETES_OF_THE_YOUNG	21	-0.57	-2.17	0.00E+00	2.36E-03	5.40E-02	Neg
REACTOME_METABOLISM_OF_STEROID_HORMONES_AND_VITAMINS_A_AND_D	30	-0.52	-2.16	3.75E-04	2.39E-03	5.77E-02	Neg
REACTOME_VOLTAGE_GATED_POTASSIUM_CHANNELS	42	-0.46	-2.11	0.00E+00	3.55E-03	8.90E-02	Neg
REACTOME_AMINE_DERIVED_HORMONES	15	-0.63	-2.11	1.27E-03	3.54E-03	9.31E-02	Neg
REACTOME_CELL_CELL_JUNCTION_ORGANIZATION	48	-0.44	-2.09	0.00E+00	3.93E-03	1.08E-01	Neg
REACTOME_STEROID_HORMONES	25	-0.52	-2.09	0.00E+00	3.75E-03	1.08E-01	Neg
REACTOME_DEPOSITION_OF_NEW_CENPA_CONTAINING_NUCLEOSOMES_AT_THE_CENTROMERE	24	-0.53	-2.08	7.03E-04	4.03E-03	1.19E-01	Neg
REACTOME_INFLUENZA_VIRAL_RNA_TRANSCRIPTION_AND_REPLICATION	75	-0.39	-2.06	0.00E+00	4.70E-03	1.43E-01	Neg
REACTOME_NEURONAL_SYSTEM	260	-0.31	-2.05	0.00E+00	4.74E-03	1.50E-01	Neg
REACTOME_SRP_DEPENDENT_COTRANSLATIONAL_PROTEIN_TARGETING_TO_MEMBRANE	81	-0.37	-1.98	0.00E+00	8.70E-03	2.66E-01	Neg
REACTOME_TIGHT_JUNCTION_INTERACTIONS	26	-0.49	-1.98	3.55E-04	8.42E-03	2.68E-01	Neg
NABA_SECRETED_FACTORS	265	-0.31	-1.96	0.00E+00	9.15E-03	2.97E-01	Neg
BIOCARTA_INFLAM_PATHWAY	24	-0.49	-1.94	2.44E-03	1.04E-02	3.41E-01	Neg
PID_ERBB_NETWORK_PATHWAY	15	-0.57	-1.94	4.30E-03	1.02E-02	3.44E-01	Neg

BIOCARTA_NKT_PATHWAY	**26**	**-0.47**	**-1.90**	**3.65E-03**	**1.32E-02**	**4.33E-01**	**Neg**
KEGG_GLYCOSPHINGOLIPID_BIOSYNTHESIS_LACTO_AND_NEOLACTO_SERIES	**22**	**-0.49**	**-1.88**	**4.06E-03**	**1.58E-02**	**5.01E-01**	**Neg**
REACTOME_CYTOCHROME_P450_ARRANGED_BY_SUBSTRATE_TYPE	**35**	**-0.43**	**-1.86**	**2.72E-03**	**1.69E-02**	**5.34E-01**	**Neg**
REACTOME_CLASS_B_2_SECRETIN_FAMILY_RECEPTORS	**79**	**-0.35**	**-1.86**	**0.00E+00**	**1.70E-02**	**5.48E-01**	**Neg**
BIOCARTA_CYTOKINE_PATHWAY	**18**	**-0.52**	**-1.86**	**5.49E-03**	**1.65E-02**	**5.48E-01**	**Neg**
REACTOME_TRANSPORT_OF_GLUCOSE_AND_OTHER_SUGARS_BILE_SALTS_AND_ORGANIC_ACIDS_METAL_IONS_AND_AMINE_COMPOUNDS	**82**	**-0.35**	**-1.86**	**0.00E+00**	**1.65E-02**	**5.57E-01**	**Neg**
PID_RHODOPSIN_PATHWAY	**22**	**-0.49**	**-1.85**	**4.16E-03**	**1.65E-02**	**5.67E-01**	**Neg**
KEGG_INTESTINAL_IMMUNE_NETWORK_FOR_IGA_PRODUCTION	**32**	**-0.43**	**-1.83**	**3.81E-03**	**1.85E-02**	**6.20E-01**	**Neg**
NABA_ECM_REGULATORS	**205**	**-0.29**	**-1.83**	**0.00E+00**	**1.88E-02**	**6.35E-01**	**Neg**
REACTOME_G1_S_SPECIFIC_TRANSCRIPTION	**16**	**-0.53**	**-1.83**	**9.40E-03**	**1.86E-02**	**6.40E-01**	**Neg**
KEGG_AUTOIMMUNE_THYROID_DISEASE	**19**	**-0.50**	**-1.81**	**7.58E-03**	**2.04E-02**	**6.83E-01**	**Neg**
REACTOME_GAP_JUNCTION_ASSEMBLY	**16**	**-0.52**	**-1.80**	**1.21E-02**	**2.19E-02**	**7.17E-01**	**Neg**
REACTOME_TRANSPORT_OF_INORGANIC_CATIONS_ANIONS_AND_AMINO_ACIDS_OLIGOPEPTIDES	**88**	**-0.33**	**-1.80**	**0.00E+00**	**2.15E-02**	**7.19E-01**	**Neg**
REACTOME_AMINE_LIGAND_BINDING_RECEPTORS	**32**	**-0.42**	**-1.79**	**3.09E-03**	**2.25E-02**	**7.42E-01**	**Neg**
REACTOME_G_ALPHA_Q_SIGNALLING_EVENTS	**158**	**-0.29**	**-1.76**	**0.00E+00**	**2.72E-02**	**8.11E-01**	**Neg**
REACTOME_GPCR_DOWNSTREAM_SIGNALING	**441**	**-0.25**	**-1.72**	**0.00E+00**	**3.46E-02**	**8.86E-01**	**Neg**
REACTOME_AMYLOIDS	**32**	**-0.40**	**-1.69**	**8.06E-03**	**4.08E-02**	**9.28E-01**	**Neg**
KEGG_GLYCOSAMINOGLYCAN_BIOSYNTHESIS_HEPARAN_SULFATE	**24**	**-0.43**	**-1.68**	**1.76E-02**	**4.38E-02**	**9.46E-01**	**Neg**
KEGG_TASTE_TRANSDUCTION	**36**	**-0.38**	**-1.67**	**6.48E-03**	**4.63E-02**	**9.56E-01**	**Neg**
REACTOME_G_ALPHA_I_SIGNALLING_EVENTS	**163**	**-0.27**	**-1.66**	**0.00E+00**	**4.68E-02**	**9.59E-01**	**Neg**
KEGG_STEROID_HORMONE_BIOSYNTHESIS	**26**	**-0.41**	**-1.65**	**1.38E-02**	**4.82E-02**	**9.65E-01**	**Neg**
REACTOME_FORMATION_OF_THE_TERNARY_COMPLEX_AND_SUBSEQUENTLY_THE_43S_COMPLEX	**36**	**-0.38**	**-1.65**	**1.12E-02**	**4.90E-02**	**9.69E-01**	**Neg**

Supplementary table 3: Gene Set Enrichment Analysis (GSEA) results of the PER3 co-expressed genes in the D1 healthy human mammary tissue dataset.

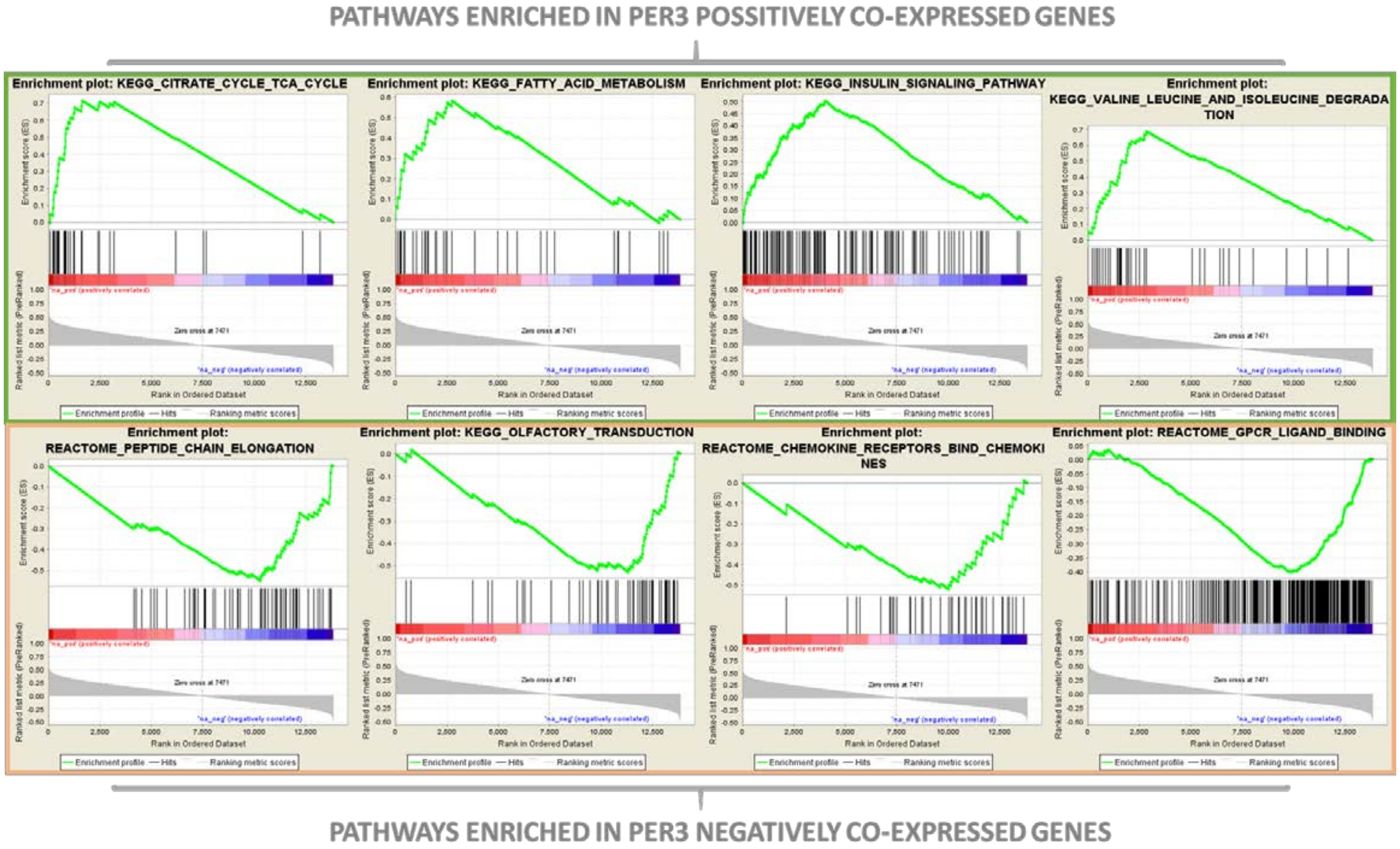

Supplementary figure 1: Top pathways enrichen in both positively and negatively PER3 co-expressed genes in the healthy breast tissue D1 dataset.

NAME	SIZE	ES	NES	NOM p-val	FDR q-val	FWER p-val	PER3 Pos-Neg
KEGG_PEROXISOME	72	0.60	2.56	0.00E+00	0.00E+00	0.00E+00	Pos
KEGG_VALINE_LEUCINE_AND_ISOLEUCINE_DEGRADATION	42	0.64	2.42	0.00E+00	3.60E-05	1.00E-04	Pos
REACTOME_BRANCHED_CHAIN_AMINO_ACID_CATABOLISM	16	0.77	2.31	0.00E+00	9.71E-05	4.00E-04	Pos
KEGG_CITRATE_CYCLE_TCA_CYCLE	29	0.66	2.28	0.00E+00	2.71E-04	1.50E-03	Pos
REACTOME_BIOLOGICAL_OXIDATIONS	88	0.51	2.25	0.00E+00	3.74E-04	2.60E-03	Pos
REACTOME_PYRUVATE_METABOLISM_AND_CITRIC_ACID_TCA_CYCLE	37	0.61	2.25	0.00E+00	3.60E-04	3.00E-03	Pos
KEGG_DRUG_METABOLISM_CYTOCHROME_P450	35	0.61	2.24	0.00E+00	3.50E-04	3.40E-03	Pos
KEGG_PROPANOATE_METABOLISM	29	0.64	2.23	0.00E+00	3.70E-04	4.10E-03	Pos
REACTOME_TCA_CYCLE_AND_RESPIRATORY_ELECTRON_TRANSPORT	90	0.50	2.22	0.00E+00	3.53E-04	4.40E-03	Pos
KEGG_OXIDATIVE_PHOSPHORYLATION	89	0.46	2.05	0.00E+00	4.91E-03	6.53E-02	Pos
REACTOME_CITRIC_ACID_CYCLE_TCA_CYCLE	18	0.66	2.04	1.67E-04	4.93E-03	7.20E-02	Pos
REACTOME_RESPIRATORY_ELECTRON_TRANSPORT	54	0.51	2.04	0.00E+00	4.54E-03	7.24E-02	Pos
KEGG_FATTY_ACID_METABOLISM	35	0.55	2.03	1.61E-04	4.80E-03	8.22E-02	Pos
KEGG_PARKINSONS_DISEASE	85	0.46	2.02	0.00E+00	5.08E-03	9.35E-02	Pos
REACTOME_SIGNALING_BY_INSULIN_RECEPTOR	102	0.44	2.00	0.00E+00	6.51E-03	1.26E-01	Pos
REACTOME_PHASE1_FUNCTIONALIZATION_OF_COMPOUNDS	51	0.49	1.97	0.00E+00	8.82E-03	1.76E-01	Pos
REACTOME_PHASE_II_CONJUGATION	38	0.53	1.96	9.75E-04	9.75E-03	2.03E-01	Pos
NABA_CORE_MATRISOME	243	0.38	1.94	1.33E-04	1.15E-02	2.49E-01	Pos
REACTOME_RESPIRATORY_ELECTRON_TRANSPORT_ATP_SYNTHESIS_BY_CHEMIOSMOTIC_COUPLING_AND_HEAT_PRODUCTION_BY_UNCOUPLING_PROTEINS_	57	0.48	1.93	3.15E-04	1.21E-02	2.72E-01	Pos
REACTOME_METABOLISM_OF_AMINO_ACIDS_AND_DERIVATIVES	174	0.40	1.93	0.00E+00	1.18E-02	2.77E-01	Pos
REACTOME_METABOLISM_OF_VITAMINS_AND_COFACTORS	47	0.49	1.92	1.59E-04	1.22E-02	2.96E-01	Pos
REACTOME_INSULIN_RECEPTOR_SIGNALLING_CASCADE	82	0.44	1.92	1.52E-04	1.19E-02	3.04E-01	Pos
NABA_ECM_GLYCOPROTEINS	169	0.39	1.91	0.00E+00	1.26E-02	3.29E-01	Pos
KEGG_RENIN_ANGIOTENSIN_SYSTEM	17	0.63	1.90	1.89E-03	1.47E-02	3.89E-01	Pos
PID_BMP_PATHWAY	40	0.50	1.89	4.82E-04	1.62E-02	4.32E-01	Pos
REACTOME_PEROXISOMAL_LIPID_METABOLISM	20	0.59	1.88	1.85E-03	1.66E-02	4.54E-01	Pos
BIOCARTA_PROTEASOME_PATHWAY	27	0.55	1.87	1.50E-03	1.70E-02	4.75E-01	Pos
REACTOME_PI3K_CASCADE	67	0.45	1.86	0.00E+00	1.89E-02	5.25E-01	Pos
KEGG_METABOLISM_OF_XENOBIOTICS_BY_CYTOCHROME_P450	33	0.51	1.82	1.80E-03	2.84E-02	6.81E-01	Pos
KEGG_TGF_BETA_SIGNALING_PATHWAY	79	0.42	1.80	1.51E-04	3.29E-02	7.45E-01	Pos
REACTOME_PYRUVATE_METABOLISM	16	0.60	1.79	4.62E-03	3.65E-02	7.90E-01	Pos
KEGG_HISTIDINE_METABOLISM	26	0.52	1.79	4.17E-03	3.67E-02	8.01E-01	Pos
REACTOME_GLUCOSE_METABOLISM	58	0.43	1.77	1.25E-03	4.18E-02	8.53E-01	Pos
NABA_ECM_REGULATORS	205	0.35	1.77	0.00E+00	4.11E-02	8.57E-01	Pos
KEGG_GLYCOLYSIS_GLUCONEOGENESIS	53	0.44	1.77	2.04E-03	4.09E-02	8.64E-01	Pos
REACTOME_SIGNALING_BY_BMP	22	0.54	1.77	5.04E-03	4.08E-02	8.71E-01	Pos

REACTOME_BIOSYNTHESIS_OF_THE_N_GLYCAN_PRECURSOR_DOLICHOL_LIPID_LINKED_OLIGOSACCHARIDE_LLO_AND_TRANSFER_TO_A_NASCENT_PROTEIN	21	0.55	1.76	5.17E-03	4.29E-02	8.91E-01	Pos
KEGG_STEROID_HORMONE_BIOSYNTHESIS	26	0.51	1.73	4.92E-03	5.81E-02	9.57E-01	Pos
REACTOME_ABC_FAMILY_PROTEINS_MEDIATED_TRANSPORT	31	0.49	1.72	6.82E-03	5.79E-02	9.60E-01	Pos
PID_INSULIN_GLUCOSE_PATHWAY	26	0.51	1.72	7.07E-03	5.72E-02	9.61E-01	Pos
KEGG_PYRUVATE_METABOLISM	37	0.46	1.72	4.19E-03	5.91E-02	9.68E-01	Pos
KEGG_PPAR_SIGNALING_PATHWAY	64	0.41	1.72	2.46E-03	5.85E-02	9.69E-01	Pos
REACTOME_COLLAGEN_FORMATION	52	0.43	1.71	2.68E-03	6.22E-02	9.77E-01	Pos
KEGG_ARACHIDONIC_ACID_METABOLISM	45	0.43	1.68	5.43E-03	7.88E-02	9.92E-01	Pos
REACTOME_CYTOCHROME_P450_ARRANGED_BY_SUBSTRATE_TYPE	35	0.46	1.68	5.52E-03	7.82E-02	9.92E-01	Pos
KEGG_PROTEASOME	40	0.44	1.67	6.73E-03	7.71E-02	9.93E-01	Pos
BIOCARTA_CHREBP2_PATHWAY	39	0.45	1.67	6.77E-03	7.75E-02	9.94E-01	Pos
KEGG_LYSOSOME	110	0.36	1.65	7.40E-04	8.98E-02	9.98E-01	Pos
KEGG_GLYCINE_SERINE_AND_THREONINE_METABOLISM	31	0.46	1.65	8.69E-03	9.31E-02	9.99E-01	Pos
REACTOME_FGFR_LIGAND_BINDING_AND_ACTIVATION	22	0.51	1.65	1.58E-02	9.25E-02	9.99E-01	Pos
REACTOME_ABCA_TRANSPORTERS_IN_LIPID_HOMEOSTASIS	16	0.55	1.64	1.70E-02	9.21E-02	9.99E-01	Pos
NABA_PROTEOGLYCANS	34	0.45	1.64	1.19E-02	9.05E-02	9.99E-01	Pos
KEGG_COMPLEMENT_AND_COAGULATION_CASCADES	60	0.40	1.64	4.91E-03	9.01E-02	9.99E-01	Pos
KEGG_REGULATION_OF_AUTOPHAGY	21	0.51	1.64	1.42E-02	8.85E-02	9.99E-01	Pos
KEGG_PROTEIN_EXPORT	21	0.51	1.63	1.61E-02	9.28E-02	1.00E+00	Pos
REACTOME_IRON_UPTAKE_AND_TRANSPORT	35	0.45	1.63	1.31E-02	9.35E-02	1.00E+00	Pos
REACTOME_REGULATION_OF_ORNITHINE_DECARBOXYLASE_ODC	45	0.42	1.62	7.79E-03	9.92E-02	1.00E+00	Pos
REACTOME_INSULIN_RECEPTOR_RECYCLING	22	0.50	1.62	2.22E-02	1.02E-01	1.00E+00	Pos
KEGG_BUTANOATE_METABOLISM	31	0.45	1.61	1.42E-02	1.03E-01	1.00E+00	Pos
REACTOME_PKB_MEDIATED_EVENTS	27	0.47	1.61	1.65E-02	1.01E-01	1.00E+00	Pos
REACTOME_SIGNALING_BY_WNT	56	0.39	1.60	7.64E-03	1.07E-01	1.00E+00	Pos
REACTOME_ANTIGEN_PROCESSING_UBIQUITINATION_PROTEASOME_DEGRADATION	179	0.32	1.60	9.69E-04	1.06E-01	1.00E+00	Pos
REACTOME_CROSS_PRESENTATION_OF_SOLUBLE_EXOGENOUS_ANTIGENS_ENDOSOMES	43	0.42	1.60	1.49E-02	1.05E-01	1.00E+00	Pos
KEGG_PRIMARY_BILE_ACID_BIOSYNTHESIS	16	0.54	1.60	2.30E-02	1.05E-01	1.00E+00	Pos
BIOCARTA_IGF1MTOR_PATHWAY	19	0.51	1.59	2.42E-02	1.16E-01	1.00E+00	Pos
KEGG_BETA_ALANINE_METABOLISM	20	0.50	1.58	2.78E-02	1.21E-01	1.00E+00	Pos
REACTOME_ACTIVATED_POINT_MUTANTS_OF_FGFR2	16	0.53	1.58	3.15E-02	1.20E-01	1.00E+00	Pos
KEGG_ABC_TRANSPORTERS	41	0.42	1.58	1.53E-02	1.21E-01	1.00E+00	Pos
REACTOME_GLYCEROPHOSPHOLIPID_BIOSYNTHESIS	77	0.36	1.56	8.31E-03	1.33E-01	1.00E+00	Pos
PID_INTEGRIN1_PATHWAY	63	0.37	1.55	1.39E-02	1.42E-01	1.00E+00	Pos
PID_NCADHERIN_PATHWAY	31	0.44	1.55	2.89E-02	1.43E-01	1.00E+00	Pos
PID_A6B1_A6B4_INTEGRIN_PATHWAY	44	0.40	1.54	1.94E-02	1.46E-01	1.00E+00	Pos
REACTOME_EXTRACELLULAR_MATRIX_ORGANIZATION	75	0.36	1.54	9.12E-03	1.47E-01	1.00E+00	Pos
BIOCARTA_CK1_PATHWAY	16	0.51	1.53	4.07E-02	1.54E-01	1.00E+00	Pos
REACTOME_CLASS_I_MHC_MEDIATED_ANTIGEN_PROCESSING_PRESENTATION	210	0.31	1.53	1.38E-03	1.52E-01	1.00E+00	Pos
KEGG_TYROSINE_METABOLISM	37	0.41	1.53	2.48E-02	1.57E-01	1.00E+00	Pos
REACTOME_TRANSFERRIN_ENDOCYTOSIS_AND_RECYCLING	24	0.46	1.52	4.05E-02	1.64E-01	1.00E+00	Pos

REACTOME_LIPID_DIGESTION_MOBILIZATION_AND_TRANSPORT	43	0.40	1.52	2.39E-02	1.62E-01	1.00E+00	Pos
REACTOME_FRS2_MEDIATED_CASCADE	35	0.42	1.52	3.14E-02	1.62E-01	1.00E+00	Pos
KEGG_ALZHEIMERS_DISEASE	130	0.32	1.52	6.21E-03	1.64E-01	1.00E+00	Pos
KEGG_NICOTINATE_AND_NICOTINAMIDE_METABOLISM	20	0.48	1.51	4.21E-02	1.64E-01	1.00E+00	Pos
KEGG_SELENOAMINO_ACID_METABOLISM	23	0.46	1.51	4.27E-02	1.66E-01	1.00E+00	Pos
KEGG_GLUTATHIONE_METABOLISM	39	0.40	1.51	2.87E-02	1.67E-01	1.00E+00	Pos
REACTOME_ASSOCIATION_OF_TRIC_CCT_WITH_TARGET_PROTEINS_DURING_BIOSYNTHESIS	23	0.46	1.51	4.03E-02	1.67E-01	1.00E+00	Pos
REACTOME_PEPTIDE_CHAIN_ELONGATION	60	-0.75	-3.38	0.00E+00	0.00E+00	0.00E+00	Neg
KEGG_RIBOSOME	63	-0.72	-3.24	0.00E+00	0.00E+00	0.00E+00	Neg
REACTOME_3_UTR_MEDIATED_TRANSLATIONAL_REGULATION	74	-0.67	-3.13	0.00E+00	0.00E+00	0.00E+00	Neg
REACTOME_NONSENSE_MEDIATED_DECAY_ENHANCED_BY_THE_EXON_JUNCTION_COMPLEX	75	-0.64	-3.02	0.00E+00	0.00E+00	0.00E+00	Neg
REACTOME_INFLUENZA_VIRAL_RNA_TRANSCRIPTION_AND_REPLICATION	75	-0.64	-3.00	0.00E+00	0.00E+00	0.00E+00	Neg
KEGG_PRIMARY_IMMUNODEFICIENCY	34	-0.76	-2.99	0.00E+00	0.00E+00	0.00E+00	Neg
PID_CD8_TCR_PATHWAY	51	-0.68	-2.94	0.00E+00	0.00E+00	0.00E+00	Neg
PID_TCR_PATHWAY	60	-0.65	-2.91	0.00E+00	0.00E+00	0.00E+00	Neg
REACTOME_TELOMERE_MAINTENANCE	40	-0.70	-2.83	0.00E+00	0.00E+00	0.00E+00	Neg
REACTOME_INFLUENZA_LIFE_CYCLE	105	-0.57	-2.83	0.00E+00	0.00E+00	0.00E+00	Neg
REACTOME_GENERATION_OF_SECOND_MESSENGER_MOLECULES	19	-0.83	-2.79	0.00E+00	0.00E+00	0.00E+00	Neg
REACTOME_SRP_DEPENDENT_COTRANSLATIONAL_PROTEIN_TARGETING_TO_MEMBRANE	81	-0.58	-2.77	0.00E+00	0.00E+00	0.00E+00	Neg
PID_IL12_2PATHWAY	55	-0.62	-2.72	0.00E+00	0.00E+00	0.00E+00	Neg
REACTOME_EXTENSION_OF_TELOMERES	25	-0.73	-2.67	0.00E+00	0.00E+00	0.00E+00	Neg
REACTOME_TCR_SIGNALING	43	-0.64	-2.65	0.00E+00	0.00E+00	0.00E+00	Neg
REACTOME_PROCESSING_OF_CAPPED_INTRON_CONTAINING_PRE_MRNA	113	-0.52	-2.63	0.00E+00	0.00E+00	0.00E+00	Neg
REACTOME_IMMUNOREGULATORY_INTERACTIONS_BETWEEN_A_LYMPHOID_AND_A_NON_LYMPHOID_CELL	32	-0.68	-2.63	0.00E+00	0.00E+00	0.00E+00	Neg
REACTOME_FORMATION_OF_THE_TERNARY_COMPLEX_AND_SUBSEQUENTLY_THE_43S_COMPLEX	36	-0.66	-2.62	0.00E+00	0.00E+00	0.00E+00	Neg
KEGG_INTESTINAL_IMMUNE_NETWORK_FOR_IGA_PRODUCTION	32	-0.67	-2.59	0.00E+00	0.00E+00	0.00E+00	Neg
PID_IL27_PATHWAY	26	-0.71	-2.58	0.00E+00	0.00E+00	0.00E+00	Neg
PID_BCR_5PATHWAY	61	-0.57	-2.56	0.00E+00	6.92E-06	1.00E-04	Neg
KEGG_T_CELL_RECEPTOR_SIGNALING_PATHWAY	102	-0.52	-2.56	0.00E+00	6.61E-06	1.00E-04	Neg
KEGG_DNA_REPLICATION	34	-0.65	-2.54	0.00E+00	6.32E-06	1.00E-04	Neg
REACTOME_ACTIVATION_OF_ATR_IN_RESPONSE_TO_REPLICATION_STRESS	27	-0.68	-2.53	0.00E+00	6.06E-06	1.00E-04	Neg

REACTOME_CHEMOKINE_RECEPTORS_BIND_CHEMOKINES	**38**	**-0.62**	**-2.50**	**0.00E+00**	**1.79E-05**	**3.00E-04**	**Neg**
REACTOME_ACTIVATION_OF_THE_PRE_REPLICATIVE_COMPLEX	**23**	**-0.71**	**-2.49**	**0.00E+00**	**1.72E-05**	**3.00E-04**	**Neg**
REACTOME_MRNA_SPLICING	**87**	**-0.52**	**-2.49**	**0.00E+00**	**1.66E-05**	**3.00E-04**	**Neg**
REACTOME_METABOLISM_OF_RNA	**208**	**-0.44**	**-2.46**	**0.00E+00**	**3.19E-05**	**6.00E-04**	**Neg**
REACTOME_METABOLISM_OF_MRNA	**169**	**-0.46**	**-2.46**	**0.00E+00**	**3.08E-05**	**6.00E-04**	**Neg**
BIOCARTA_NO2IL12_PATHWAY	**16**	**-0.78**	**-2.46**	**0.00E+00**	**2.98E-05**	**6.00E-04**	**Neg**
PID_FANCONI_PATHWAY	**40**	**-0.61**	**-2.44**	**0.00E+00**	**4.74E-05**	**1.00E-03**	**Neg**
ST_T_CELL_SIGNAL_TRANSDUCTION	**42**	**-0.59**	**-2.43**	**0.00E+00**	**4.59E-05**	**1.00E-03**	**Neg**
KEGG_B_CELL_RECEPTOR_SIGNALING_PATHWAY	**67**	**-0.53**	**-2.42**	**0.00E+00**	**5.73E-05**	**1.30E-03**	**Neg**
BIOCARTA_TNFR2_PATHWAY	**17**	**-0.74**	**-2.40**	**0.00E+00**	**6.44E-05**	**1.50E-03**	**Neg**
REACTOME_G2_M_CHECKPOINTS	**33**	**-0.61**	**-2.40**	**0.00E+00**	**7.08E-05**	**1.70E-03**	**Neg**
REACTOME_DNA_STRAND_ELONGATION	**29**	**-0.64**	**-2.39**	**0.00E+00**	**7.29E-05**	**1.80E-03**	**Neg**
REACTOME_TRANSLATION	**110**	**-0.47**	**-2.38**	**0.00E+00**	**9.10E-05**	**2.30E-03**	**Neg**
REACTOME_ANTIGEN_ACTIVATES_B_CELL_RECEPTOR_LEADING_TO_GENERATION_OF_SECOND_MESSENGERS	**27**	**-0.64**	**-2.38**	**0.00E+00**	**8.86E-05**	**2.30E-03**	**Neg**
REACTOME_LAGGING_STRAND_SYNTHESIS	**18**	**-0.72**	**-2.36**	**0.00E+00**	**9.73E-05**	**2.60E-03**	**Neg**
PID_NFAT_TFPATHWAY	**44**	**-0.57**	**-2.36**	**0.00E+00**	**1.02E-04**	**2.80E-03**	**Neg**
PID_CD8_TCR_DOWNSTREAM_PATHWAY	**50**	**-0.55**	**-2.35**	**0.00E+00**	**1.18E-04**	**3.30E-03**	**Neg**
PID_IL12_STAT4_PATHWAY	**30**	**-0.62**	**-2.34**	**0.00E+00**	**1.18E-04**	**3.40E-03**	**Neg**
KEGG_HOMOLOGOUS_RECOMBINATION	**22**	**-0.67**	**-2.33**	**0.00E+00**	**1.35E-04**	**4.00E-03**	**Neg**
KEGG_LEISHMANIA_INFECTION	**52**	**-0.53**	**-2.30**	**0.00E+00**	**1.72E-04**	**5.20E-03**	**Neg**
REACTOME_ACTIVATION_OF_THE_MRNA_UPON_BINDING_OF_THE_CAP_BINDING_COMPLEX_AND_EIFS_AND_SUBSEQUENT_BINDING_TO_43S	**42**	**-0.56**	**-2.30**	**0.00E+00**	**1.68E-04**	**5.20E-03**	**Neg**
REACTOME_MRNA_PROCESSING	**130**	**-0.44**	**-2.29**	**0.00E+00**	**1.97E-04**	**6.20E-03**	**Neg**
REACTOME_FANCONI_ANEMIA_PATHWAY	**19**	**-0.69**	**-2.29**	**0.00E+00**	**1.95E-04**	**6.30E-03**	**Neg**
KEGG_SPLICEOSOME	**99**	**-0.46**	**-2.27**	**0.00E+00**	**2.42E-04**	**8.00E-03**	**Neg**
REACTOME_MEIOTIC_RECOMBINATION	**35**	**-0.57**	**-2.27**	**0.00E+00**	**2.37E-04**	**8.00E-03**	**Neg**
BIOCARTA_IL22BP_PATHWAY	**16**	**-0.71**	**-2.25**	**0.00E+00**	**3.05E-04**	**1.05E-02**	**Neg**
REACTOME_INTERFERON_GAMMA_SIGNALING	**41**	**-0.55**	**-2.25**	**0.00E+00**	**3.02E-04**	**1.06E-02**	**Neg**
PID_IFNG_PATHWAY	**39**	**-0.56**	**-2.25**	**0.00E+00**	**3.01E-04**	**1.08E-02**	**Neg**

KEGG_GRAFT_VERSUS_HOST_DISEASE	15	-0.72	-2.24	0.00E+00	3.15E-04	1.14E-02	Neg
PID_IL23_PATHWAY	37	-0.56	-2.24	0.00E+00	3.49E-04	1.28E-02	Neg
KEGG_ALLOGRAFT_REJECTION	17	-0.68	-2.22	0.00E+00	4.49E-04	1.68E-02	Neg
REACTOME_DEPOSITION_OF_NEW_CENPA_CONTAINING_NUCLEOSOMES_AT_THE_CENTROMERE	24	-0.62	-2.21	2.49E-04	4.72E-04	1.80E-02	Neg
PID_PI3KCI_PATHWAY	46	-0.53	-2.21	0.00E+00	4.76E-04	1.84E-02	Neg
REACTOME_CHROMOSOME_MAINTENANCE	77	-0.47	-2.18	0.00E+00	6.45E-04	2.53E-02	Neg
KEGG_CELL_ADHESION_MOLECULES_CAMS	104	-0.44	-2.18	0.00E+00	6.42E-04	2.56E-02	Neg
REACTOME_HOMOLOGOUS_RECOMBINATION_REPAIR_OF_REPLICATION_INDEPENDENT_DOUBLE_STRAND_BREAKS	15	-0.70	-2.18	2.43E-04	6.50E-04	2.64E-02	Neg
PID_ATR_PATHWAY	37	-0.55	-2.17	0.00E+00	7.06E-04	2.92E-02	Neg
BIOCARTA_IL7_PATHWAY	17	-0.67	-2.17	0.00E+00	7.87E-04	3.30E-02	Neg
REACTOME_GROWTH_HORMONE_RECEPTOR_SIGNALING	22	-0.62	-2.16	0.00E+00	7.95E-04	3.38E-02	Neg
KEGG_HEMATOPOIETIC_CELL_LINEAGE	72	-0.47	-2.16	0.00E+00	8.12E-04	3.51E-02	Neg
PID_MYC_ACTIV_PATHWAY	71	-0.47	-2.16	0.00E+00	8.33E-04	3.65E-02	Neg
KEGG_CYTOKINE_CYTOKINE_RECEPTOR_INTERACTION	212	-0.39	-2.15	0.00E+00	8.47E-04	3.77E-02	Neg
REACTOME_TRANSPORT_OF_MATURE_TRANSCRIPT_TO_CYTOPLASM	39	-0.52	-2.11	0.00E+00	1.34E-03	6.00E-02	Neg
KEGG_CHEMOKINE_SIGNALING_PATHWAY	159	-0.40	-2.11	0.00E+00	1.35E-03	6.11E-02	Neg
REACTOME_DOUBLE_STRAND_BREAK_REPAIR	21	-0.61	-2.11	2.51E-04	1.40E-03	6.41E-02	Neg
REACTOME_CLEAVAGE_OF_GROWING_TRANSCRIPT_IN_THE_TERMINATION_REGION_	31	-0.55	-2.10	0.00E+00	1.39E-03	6.47E-02	Neg
KEGG_NATURAL_KILLER_CELL_MEDIATED_CYTOTOXICITY	85	-0.44	-2.10	0.00E+00	1.43E-03	6.71E-02	Neg
PID_BARD1_PATHWAY	27	-0.57	-2.10	2.55E-04	1.49E-03	7.07E-02	Neg
REACTOME_RNA_POL_I_PROMOTER_OPENING	15	-0.68	-2.10	2.41E-04	1.49E-03	7.16E-02	Neg
BIOCARTA_TH1TH2_PATHWAY	17	-0.65	-2.09	4.89E-04	1.58E-03	7.71E-02	Neg
REACTOME_DNA_REPAIR	96	-0.43	-2.09	0.00E+00	1.57E-03	7.75E-02	Neg
PID_PLK1_PATHWAY	40	-0.51	-2.09	5.30E-04	1.66E-03	8.30E-02	Neg
REACTOME_PACKAGING_OF_TELOMERE_ENDS	15	-0.67	-2.09	2.49E-04	1.64E-03	8.30E-02	Neg
REACTOME_DNA_REPLICATION	167	-0.39	-2.09	0.00E+00	1.64E-03	8.40E-02	Neg
BIOCARTA_IL12_PATHWAY	20	-0.61	-2.06	2.52E-04	2.15E-03	1.10E-01	Neg
PID_HDAC_CLASSI_PATHWAY	62	-0.46	-2.06	0.00E+00	2.12E-03	1.10E-01	Neg

PID_TXA2PATHWAY	53	-0.47	-2.05	0.00E+00	2.37E-03	1.24E-01	Neg
BIOCARTA_NTHI_PATHWAY	22	-0.59	-2.04	7.38E-04	2.38E-03	1.26E-01	Neg
BIOCARTA_ATRBRCA_PATHWAY	18	-0.62	-2.04	7.28E-04	2.38E-03	1.27E-01	Neg
KEGG_CYTOSOLIC_DNA_SENSING_PATHWAY	38	-0.50	-2.04	0.00E+00	2.49E-03	1.34E-01	Neg
REACTOME_CYTOKINE_SIGNALING_IN_IMMUNE_SYSTEM	213	-0.37	-2.03	0.00E+00	2.58E-03	1.41E-01	Neg
PID_CD40_PATHWAY	31	-0.53	-2.03	0.00E+00	2.61E-03	1.43E-01	Neg
REACTOME_BASE_EXCISION_REPAIR	18	-0.61	-2.03	7.34E-04	2.69E-03	1.49E-01	Neg
BIOCARTA_NKT_PATHWAY	26	-0.55	-2.03	4.99E-04	2.66E-03	1.49E-01	Neg
BIOCARTA_HIVNEF_PATHWAY	53	-0.47	-2.02	0.00E+00	2.68E-03	1.51E-01	Neg
KEGG_AUTOIMMUNE_THYROID_DISEASE	19	-0.61	-2.02	1.24E-03	2.68E-03	1.53E-01	Neg
ST_TUMOR_NECROSIS_FACTOR_PATHWAY	28	-0.55	-2.02	2.55E-04	2.65E-03	1.53E-01	Neg
BIOCARTA_CSK_PATHWAY	19	-0.61	-2.02	1.21E-03	2.76E-03	1.60E-01	Neg
REACTOME_MITOTIC_PROMETAPHASE	76	-0.43	-2.02	0.00E+00	2.78E-03	1.62E-01	Neg
REACTOME_INTERFERON_SIGNALING	111	-0.40	-2.02	0.00E+00	2.83E-03	1.67E-01	Neg
PID_IL2_STAT5_PATHWAY	29	-0.54	-2.01	1.03E-03	2.83E-03	1.68E-01	Neg
KEGG_ANTIGEN_PROCESSING_AND_PRESENTATION	33	-0.52	-2.01	7.71E-04	2.91E-03	1.75E-01	Neg
BIOCARTA_CHEMICAL_PATHWAY	22	-0.58	-2.01	1.75E-03	2.99E-03	1.80E-01	Neg
BIOCARTA_G2_PATHWAY	24	-0.56	-2.01	7.61E-04	2.97E-03	1.81E-01	Neg
BIOCARTA_IL2RB_PATHWAY	36	-0.50	-2.00	0.00E+00	3.03E-03	1.86E-01	Neg
BIOCARTA_CTLA4_PATHWAY	17	-0.61	-1.99	1.72E-03	3.50E-03	2.13E-01	Neg
REACTOME_IL_3_5_AND_GM_CSF_SIGNALING	39	-0.49	-1.99	5.35E-04	3.49E-03	2.15E-01	Neg
REACTOME_DOWNSTREAM_TCR_SIGNALING	27	-0.54	-1.99	1.02E-03	3.55E-03	2.20E-01	Neg
PID_CXCR4_PATHWAY	97	-0.41	-1.99	0.00E+00	3.54E-03	2.21E-01	Neg
BIOCARTA_PML_PATHWAY	16	-0.62	-1.99	7.38E-04	3.51E-03	2.21E-01	Neg
BIOCARTA_G1_PATHWAY	27	-0.54	-1.98	5.03E-04	3.68E-03	2.32E-01	Neg
REACTOME_MRNA_3_END_PROCESSING	22	-0.57	-1.98	1.51E-03	3.76E-03	2.39E-01	Neg
KEGG_MISMATCH_REPAIR	20	-0.58	-1.97	7.46E-04	3.78E-03	2.42E-01	Neg
PID_FOXM1_PATHWAY	38	-0.48	-1.96	0.00E+00	4.31E-03	2.72E-01	Neg

KEGG_RNA_DEGRADATION	53	-0.45	-1.96	5.51E-04	4.43E-03	2.79E-01	Neg
REACTOME_COSTIMULATION_BY_THE_CD28_FAMILY	55	-0.45	-1.96	0.00E+00	4.39E-03	2.79E-01	Neg
SIG_BCR_SIGNALING_PATHWAY	43	-0.47	-1.95	8.09E-04	4.41E-03	2.83E-01	Neg
REACTOME_INTERFERON_ALPHA_BETA_SIGNALING	40	-0.48	-1.94	2.72E-04	5.19E-03	3.26E-01	Neg
REACTOME_TRANSPORT_OF_MATURE_MRNA_DERIVED_FROM_AN_INTRONLESS_TRANSCRIPT	29	-0.51	-1.94	1.50E-03	5.23E-03	3.30E-01	Neg
KEGG_TYPE_I_DIABETES_MELLITUS	23	-0.55	-1.93	3.04E-03	5.38E-03	3.40E-01	Neg
KEGG_FC_GAMMA_R_MEDIATED_PHAGOCYTOSIS	83	-0.40	-1.92	2.93E-04	5.95E-03	3.71E-01	Neg
REACTOME_MITOTIC_M_M_G1_PHASES	148	-0.36	-1.92	0.00E+00	6.00E-03	3.77E-01	Neg
PID_HIV_NEF_PATHWAY	35	-0.49	-1.92	8.00E-04	5.98E-03	3.79E-01	Neg
BIOCARTA_NFKB_PATHWAY	23	-0.55	-1.92	1.26E-03	5.98E-03	3.82E-01	Neg
BIOCARTA_STRESS_PATHWAY	24	-0.54	-1.91	1.51E-03	6.18E-03	3.95E-01	Neg
PID_REG_GR_PATHWAY	75	-0.41	-1.91	0.00E+00	6.15E-03	3.96E-01	Neg
REACTOME_DEADENYLATION_DEPENDENT_MRNA_DECAY	39	-0.47	-1.91	8.05E-04	6.11E-03	3.96E-01	Neg
BIOCARTA_IL10_PATHWAY	16	-0.60	-1.91	2.18E-03	6.09E-03	3.98E-01	Neg
SIG_PIP3_SIGNALING_IN_B_LYMPHOCYTES	33	-0.49	-1.91	1.04E-03	6.21E-03	4.05E-01	Neg
SA_CASPASE_CASCADE	17	-0.60	-1.91	2.94E-03	6.23E-03	4.09E-01	Neg
KEGG_JAK_STAT_SIGNALING_PATHWAY	131	-0.37	-1.91	0.00E+00	6.27E-03	4.13E-01	Neg
PID_ATM_PATHWAY	30	-0.50	-1.90	7.76E-04	6.34E-03	4.18E-01	Neg
PID_SMAD2_3NUCLEAR_PATHWAY	76	-0.41	-1.90	0.00E+00	6.44E-03	4.26E-01	Neg
ST_FAS_SIGNALING_PATHWAY	59	-0.43	-1.90	0.00E+00	6.67E-03	4.41E-01	Neg
REACTOME_TRAF6_MEDIATED_IRF7_ACTIVATION	18	-0.58	-1.90	3.14E-03	6.67E-03	4.44E-01	Neg
BIOCARTA_CASPASE_PATHWAY	21	-0.55	-1.89	3.97E-03	6.86E-03	4.56E-01	Neg
KEGG_TOLL_LIKE_RECEPTOR_SIGNALING_PATHWAY	83	-0.40	-1.89	0.00E+00	6.86E-03	4.59E-01	Neg
KEGG_LEUKOCYTE_TRANSENDOTHELIAL_MIGRATION	105	-0.38	-1.89	0.00E+00	6.83E-03	4.60E-01	Neg
REACTOME_TRANSCRIPTION	130	-0.37	-1.89	0.00E+00	6.89E-03	4.65E-01	Neg
PID_TNF_PATHWAY	44	-0.45	-1.88	1.90E-03	7.63E-03	5.04E-01	Neg
PID_P73PATHWAY	72	-0.41	-1.87	2.91E-04	7.91E-03	5.18E-01	Neg
BIOCARTA_PITX2_PATHWAY	15	-0.60	-1.87	3.61E-03	7.94E-03	5.21E-01	Neg

REACTOME_CELL_CYCLE	327	-0.32	-1.87	0.00E+00	8.04E-03	5.30E-01	Neg
PID_EPHA_FWDPATHWAY	34	-0.48	-1.85	2.58E-03	9.40E-03	5.90E-01	Neg
BIOCARTA_TCR_PATHWAY	42	-0.45	-1.85	1.34E-03	9.69E-03	6.03E-01	Neg
REACTOME_THE_ROLE_OF_NEF_IN_HIV1_REPLICATION_AND_DISEASE_PATHOGENESIS	26	-0.51	-1.85	2.54E-03	9.80E-03	6.11E-01	Neg
REACTOME_NEP_NS2_INTERACTS_WITH_THE_CELLULAR_EXPORT_MACHINERY	24	-0.52	-1.84	4.53E-03	1.04E-02	6.34E-01	Neg
ST_B_CELL_ANTIGEN_RECEPTOR	36	-0.46	-1.83	1.83E-03	1.07E-02	6.46E-01	Neg
REACTOME_REGULATION_OF_GLUCOKINASE_BY_GLUCOKINASE_REGULATORY_PROTEIN	24	-0.51	-1.83	3.33E-03	1.06E-02	6.48E-01	Neg
KEGG_RIG_I_LIKE_RECEPTOR_SIGNALING_PATHWAY	54	-0.42	-1.83	0.00E+00	1.06E-02	6.48E-01	Neg
PID_IL8_CXCR2_PATHWAY	32	-0.48	-1.83	2.58E-03	1.07E-02	6.57E-01	Neg
KEGG_CELL_CYCLE	113	-0.36	-1.83	0.00E+00	1.09E-02	6.67E-01	Neg
PID_EPO_PATHWAY	33	-0.47	-1.83	1.32E-03	1.09E-02	6.69E-01	Neg
KEGG_BASE_EXCISION_REPAIR	31	-0.48	-1.83	2.26E-03	1.09E-02	6.72E-01	Neg
PID_AMB2_NEUTROPHILS_PATHWAY	40	-0.45	-1.82	1.07E-03	1.11E-02	6.82E-01	Neg
PID_AURORA_B_PATHWAY	38	-0.45	-1.82	1.57E-03	1.13E-02	6.91E-01	Neg
PID_IL4_2PATHWAY	60	-0.41	-1.82	2.80E-04	1.18E-02	7.07E-01	Neg
REACTOME_INTERACTIONS_OF_VPR_WITH_HOST_CELLULAR_PROTEINS	28	-0.49	-1.81	4.55E-03	1.23E-02	7.24E-01	Neg
REACTOME_RESOLUTION_OF_AP_SITES_VIA_THE_MULTIPLE_NUCLEOTIDE_PATCH_REPLACEMENT_PATHWAY	16	-0.57	-1.81	8.25E-03	1.22E-02	7.24E-01	Neg
REACTOME_TRANSPORT_OF_RIBONUCLEOPROTEINS_INTO_THE_HOST_NUCLEUS	24	-0.50	-1.81	4.70E-03	1.23E-02	7.28E-01	Neg
BIOCARTA_INFLAM_PATHWAY	24	-0.51	-1.81	6.11E-03	1.24E-02	7.34E-01	Neg
BIOCARTA_LAIR_PATHWAY	16	-0.56	-1.80	1.04E-02	1.29E-02	7.50E-01	Neg
REACTOME_RIG_I_MDA5_MEDIATED_INDUCTION_OF_IFN_ALPHA_BETA_PATHWAYS	53	-0.41	-1.80	1.13E-03	1.32E-02	7.60E-01	Neg
REACTOME_CELL_CYCLE_MITOTIC	275	-0.31	-1.79	0.00E+00	1.34E-02	7.67E-01	Neg
PID_NFKAPPAB_CANONICAL_PATHWAY	23	-0.51	-1.79	7.08E-03	1.38E-02	7.79E-01	Neg
KEGG_NOD_LIKE_RECEPTOR_SIGNALING_PATHWAY	52	-0.42	-1.79	1.96E-03	1.38E-02	7.79E-01	Neg
REACTOME_HIV_LIFE_CYCLE	95	-0.36	-1.78	6.13E-04	1.47E-02	8.03E-01	Neg
KEGG_SYSTEMIC_LUPUS_ERYTHEMATOSUS	53	-0.41	-1.77	8.29E-04	1.54E-02	8.18E-01	Neg
REACTOME_METABOLISM_OF_NON_CODING_RNA	42	-0.43	-1.77	2.63E-03	1.57E-02	8.26E-01	Neg
BIOCARTA_DEATH_PATHWAY	27	-0.48	-1.77	6.11E-03	1.61E-02	8.35E-01	Neg

BIOCARTA_RELA_PATHWAY	16	-0.55	-1.76	9.02E-03	1.68E-02	8.48E-01	Neg
REACTOME_SIGNALING_BY_ILS	100	-0.36	-1.76	0.00E+00	1.70E-02	8.54E-01	Neg
BIOCARTA_TOB1_PATHWAY	18	-0.53	-1.75	7.13E-03	1.79E-02	8.69E-01	Neg
PID_CMYB_PATHWAY	77	-0.37	-1.75	5.80E-04	1.80E-02	8.71E-01	Neg
BIOCARTA_TNFR1_PATHWAY	28	-0.47	-1.75	5.42E-03	1.79E-02	8.71E-01	Neg
REACTOME_SMAD2_SMAD3_SMAD4_HETEROTRIMER_REGULATES_TRANSCRIPTION	25	-0.49	-1.75	6.51E-03	1.83E-02	8.77E-01	Neg
PID_NFAT_3PATHWAY	48	-0.41	-1.74	5.22E-03	1.88E-02	8.84E-01	Neg
REACTOME_NUCLEOTIDE_BINDING_DOMAIN_LEUCINE_RICH_REPEAT_CONTAINING_RECEPTOR_NLR_SIGNALING_PATHWAYS	42	-0.42	-1.74	4.00E-03	1.97E-02	8.97E-01	Neg
PID_CASPASE_PATHWAY	47	-0.41	-1.73	3.71E-03	2.08E-02	9.12E-01	Neg
PID_INTEGRIN_CS_PATHWAY	25	-0.48	-1.73	8.73E-03	2.10E-02	9.15E-01	Neg
PID_RB_1PATHWAY	58	-0.39	-1.73	2.75E-03	2.12E-02	9.19E-01	Neg
REACTOME_GPVI_MEDIATED_ACTIVATION_CASCADE	30	-0.45	-1.72	7.92E-03	2.16E-02	9.24E-01	Neg
BIOCARTA_DC_PATHWAY	19	-0.52	-1.72	1.00E-02	2.16E-02	9.26E-01	Neg
REACTOME_PROCESSING_OF_CAPPED_INTRONLESS_PRE_MRNA	22	-0.49	-1.72	9.75E-03	2.16E-02	9.26E-01	Neg
PID_FCER1_PATHWAY	55	-0.39	-1.72	4.15E-03	2.25E-02	9.36E-01	Neg
KEGG_APOPTOSIS	77	-0.36	-1.71	1.45E-03	2.26E-02	9.38E-01	Neg
PID_AURORA_A_PATHWAY	29	-0.46	-1.71	8.61E-03	2.37E-02	9.46E-01	Neg
PID_ILK_PATHWAY	43	-0.41	-1.70	3.73E-03	2.45E-02	9.52E-01	Neg
REACTOME_GLOBAL_GENOMIC_NER_GG_NER	29	-0.45	-1.68	1.01E-02	2.85E-02	9.73E-01	Neg
REACTOME_G0_AND_EARLY_G1	22	-0.48	-1.68	9.50E-03	2.88E-02	9.74E-01	Neg
KEGG_SMALL_CELL_LUNG_CANCER	80	-0.35	-1.68	2.34E-03	2.96E-02	9.77E-01	Neg
BIOCARTA_IL1R_PATHWAY	31	-0.44	-1.67	9.48E-03	3.00E-02	9.79E-01	Neg
PID_RAC1_PATHWAY	51	-0.39	-1.67	5.16E-03	3.08E-02	9.82E-01	Neg
REACTOME_REGULATION_OF_SIGNALING_BY_CBL	17	-0.51	-1.66	2.09E-02	3.19E-02	9.85E-01	Neg
KEGG_P53_SIGNALING_PATHWAY	60	-0.37	-1.66	4.45E-03	3.36E-02	9.88E-01	Neg
PID_P53_DOWNSTREAM_PATHWAY	117	-0.33	-1.65	9.66E-04	3.50E-02	9.90E-01	Neg
REACTOME_MEIOSIS	63	-0.37	-1.64	4.90E-03	3.75E-02	9.94E-01	Neg
BIOCARTA_KERATINOCYTE_PATHWAY	43	-0.39	-1.64	9.39E-03	3.80E-02	9.95E-01	Neg

REACTOME_RIP_MEDIATED_NFKB_ACTIVATION_VIA_DAI	**16**	**-0.51**	**-1.63**	**2.30E-02**	**3.86E-02**	**9.95E-01**	**Neg**
REACTOME_RNA_POL_I_RNA_POL_III_AND_MITOCHONDRIAL_TRANSCRIPTION	**68**	**-0.36**	**-1.63**	**3.80E-03**	**3.97E-02**	**9.95E-01**	**Neg**
REACTOME_G1_S_SPECIFIC_TRANSCRIPTION	**16**	**-0.51**	**-1.63**	**2.80E-02**	**4.04E-02**	**9.96E-01**	**Neg**
REACTOME_LATE_PHASE_OF_HIV_LIFE_CYCLE	**83**	**-0.34**	**-1.61**	**2.35E-03**	**4.35E-02**	**9.97E-01**	**Neg**
SIG_CD40PATHWAYMAP	**33**	**-0.41**	**-1.61**	**1.55E-02**	**4.41E-02**	**9.97E-01**	**Neg**
BIOCARTA_ATM_PATHWAY	**19**	**-0.48**	**-1.61**	**2.51E-02**	**4.43E-02**	**9.97E-01**	**Neg**
BIOCARTA_STATHMIN_PATHWAY	**17**	**-0.50**	**-1.61**	**2.53E-02**	**4.44E-02**	**9.98E-01**	**Neg**
REACTOME_IL_RECEPTOR_SHC_SIGNALING	**24**	**-0.45**	**-1.61**	**2.05E-02**	**4.51E-02**	**9.98E-01**	**Neg**
REACTOME_SYNTHESIS_OF_DNA	**80**	**-0.34**	**-1.60**	**4.11E-03**	**4.60E-02**	**9.98E-01**	**Neg**
REACTOME_INNATE_IMMUNE_SYSTEM	**198**	**-0.29**	**-1.60**	**0.00E+00**	**4.65E-02**	**9.98E-01**	**Neg**
REACTOME_ACTIVATED_TLR4_SIGNALLING	**86**	**-0.33**	**-1.59**	**3.87E-03**	**4.86E-02**	**9.99E-01**	**Neg**
REACTOME_ANTIVIRAL_MECHANISM_BY_IFN_STIMULATED_GENES	**56**	**-0.36**	**-1.59**	**7.98E-03**	**4.92E-02**	**9.99E-01**	**Neg**

Supplementary table 4: Gene Set Enrichment Analysis (GSEA) results of the PER3 co-expressed genes in the D2 healthy murine mammary tissue dataset.

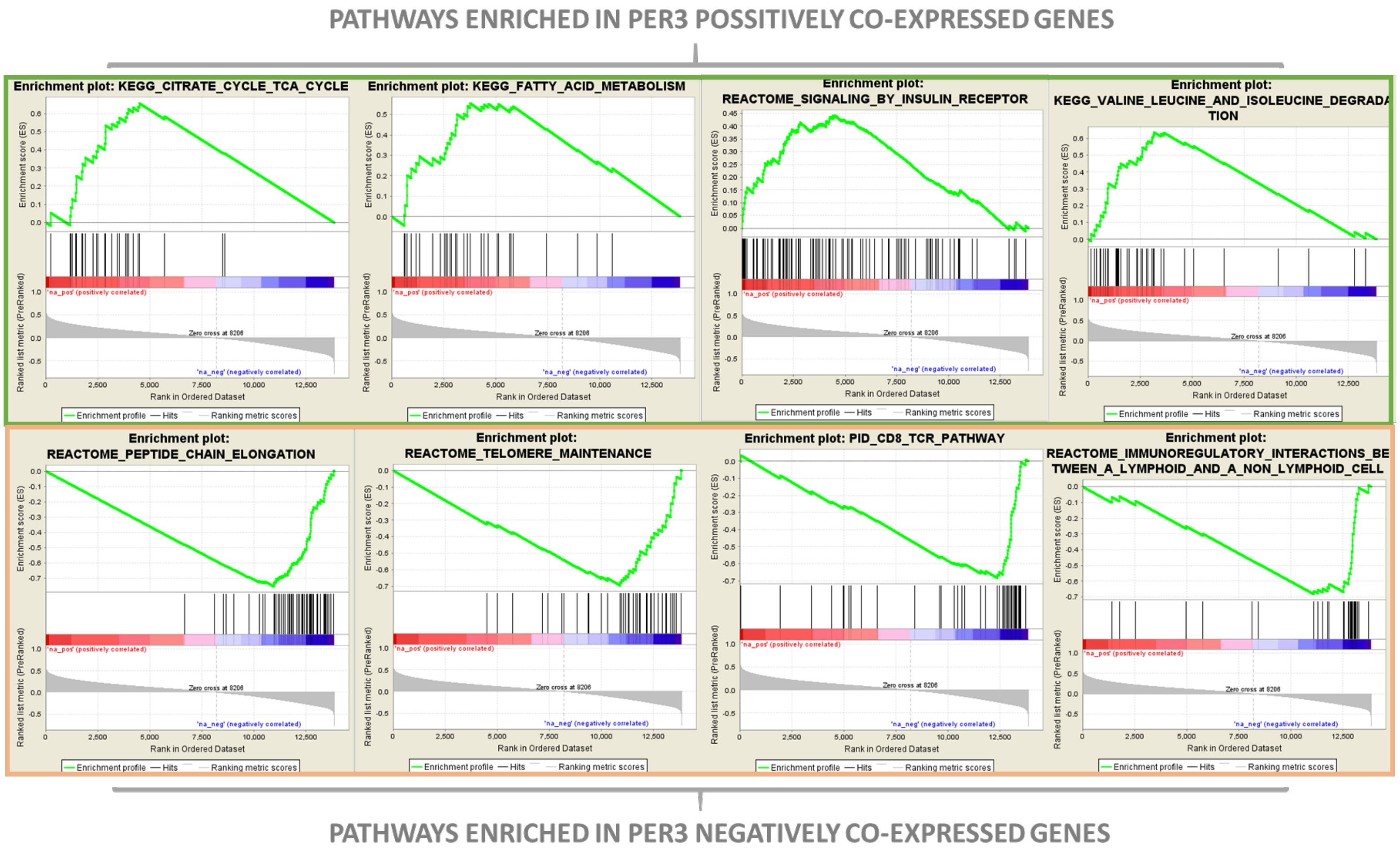

Supplementary figure 2: Top pathways enrichen in both positively and negatively PER3 co-expressed genes in the healthy breast murine tissue D2 dataset.

Array_pathway	D1 padj	D1 NES	D2 padj	D2 NES
KEGG VALINE LEUCINE AND ISOLEUCINE DEGRADATION	2.57E-04	2.59	1.26E-04	2.42
REACTOME PYRUVATE METABOLISM AND CITRIC ACID TCA CYCLE	2.57E-04	2.58	1.26E-04	2.25
KEGG CITRATE CYCLE TCA CYCLE	2.57E-04	2.49	1.26E-04	2.29
KEGG PROPANOATE METABOLISM	2.57E-04	2.41	1.26E-04	2.23
REACTOME TCA CYCLE AND RESPIRATORY ELECTRON TRANSPORT	2.57E-04	2.41	1.26E-04	2.22
REACTOME CITRIC ACID CYCLE TCA CYCLE	2.57E-04	2.33	2.70E-03	2.03
KEGG PYRUVATE METABOLISM	4.80E-04	2.19	2.56E-02	1.71
REACTOME MITOCHONDRIAL FATTY ACID BETA OXIDATION	7.51E-04	2.17	3.24E-02	1.77
KEGG FATTY ACID METABOLISM	7.06E-04	2.12	1.26E-03	2.02
REACTOME BRANCHED CHAIN AMINO ACID CATABOLISM	1.44E-03	2.11	1.26E-04	2.3
REACTOME PEROXISOMAL LIPID METABOLISM	1.07E-03	2.11	1.09E-02	1.88
KEGG PPAR SIGNALING PATHWAY	4.67E-04	2.08	1.19E-02	1.72
REACTOME RESPIRATORY ELECTRON TRANSPORT	4.80E-04	2.07	5.10E-04	2.04
REACTOME RESPIRATORY ELECTRON TRANSPORT ATP SYNTHESIS BY CHEMIOSMOTIC COUPLING AND HEAT PRODUCTION BY UNCOUPLING PROTEINS	8.39E-04	2.03	1.45E-03	1.94
REACTOME ANTIGEN PROCESSING UBIQUITINATION PROTEASOME DEGRADATION	2.57E-04	2.02	6.83E-03	1.6
REACTOME CLASS I MHC MEDIATED ANTIGEN PROCESSING PRESENTATION	2.57E-04	2.02	9.40E-03	1.54
REACTOME REGULATION OF PYRUVATE DEHYDROGENASE PDH COMPLEX	3.58E-03	2	1.91E-02	1.83
REACTOME PYRUVATE METABOLISM	4.73E-03	1.98	2.56E-02	1.79
REACTOME SIGNALING BY WNT	1.29E-03	1.98	4.03E-02	1.6
BIOCARTA PROTEASOME PATHWAY	3.69E-03	1.97	8.81E-03	1.88
REACTOME SIGNALING BY BMP	4.93E-03	1.95	2.56E-02	1.77
REACTOME REGULATION OF ORNITHINE DECARBOXYLASE ODC	2.42E-03	1.94	4.13E-02	1.63
KEGG PEROXISOME	1.51E-03	1.9	1.26E-04	2.56
REACTOME GLUCOSE METABOLISM	2.52E-03	1.9	8.96E-03	1.77
PID BMP PATHWAY	9.27E-03	1.82	4.89E-03	1.88
KEGG PARKINSONS DISEASE	4.72E-03	1.77	1.50E-04	2.02
KEGG PROTEASOME	1.63E-02	1.76	3.24E-02	1.67
REACTOME VEGF LIGAND RECEPTOR INTERACTIONS	2.90E-02	1.75	4.67E-02	1.71
BIOCARTA CHREBP2 PATHWAY	2.84E-02	1.69	3.09E-02	1.68
KEGG TGF BETA SIGNALING PATHWAY	1.42E-02	1.68	3.04E-03	1.81
KEGG LYSOSOME	8.94E-03	1.67	9.95E-03	1.65
REACTOME METABOLISM OF VITAMINS AND COFACTORS	3.94E-02	1.62	3.09E-03	1.91
KEGG GLYCOLYSIS GLUCONEOGENESIS	4.22E-02	1.59	1.00E-02	1.77
REACTOME INSULIN RECEPTOR SIGNALLING CASCADE	4.09E-02	1.54	6.25E-04	1.93
KEGG ALZHEIMERS DISEASE	3.15E-02	1.51	3.17E-02	1.51
REACTOME TRANSLATION	4.74E-02	-1.41	1.26E-04	-2.38
KEGG CYTOKINE CYTOKINE RECEPTOR INTERACTION	9.87E-03	-1.46	1.31E-04	-2.15
KEGG HEMATOPOIETIC CELL LINEAGE	3.98E-02	-1.52	2.58E-04	-2.16
REACTOME FORMATION OF THE TERNARY COMPLEX AND SUBSEQUENTLY THE 43S COMPLEX	4.12E-02	-1.65	1.26E-04	-2.61
KEGG AUTOIMMUNE THYROID DISEASE	3.36E-02	-1.81	7.04E-03	-2.02

KEGG INTESTINAL IMMUNE NETWORK FOR IGA PRODUCTION	**1.56E-02**	**-1.85**	**1.26E-04**	**-2.59**
BIOCARTA NKT PATHWAY	**1.59E-02**	**-1.9**	**4.86E-03**	**-2.02**
BIOCARTA INFLAM PATHWAY	**1.35E-02**	**-1.94**	**2.43E-02**	**-1.82**
REACTOME SRP DEPENDENT COTRANSLATIONAL PROTEIN TARGETING TO MEMBRANE	**4.80E-04**	**-1.98**	**1.26E-04**	**-2.76**
REACTOME INFLUENZA VIRAL RNA TRANSCRIPTION AND REPLICATION	**7.06E-04**	**-2.05**	**1.26E-04**	**-3**
REACTOME DEPOSITION OF NEW CENPA CONTAINING NUCLEOSOMES AT THE CENTROMERE	**6.86E-03**	**-2.07**	**5.91E-04**	**-2.22**
REACTOME NONSENSE MEDIATED DECAY ENHANCED BY THE EXON JUNCTION COMPLEX	**4.80E-04**	**-2.18**	**1.26E-04**	**-3.01**
REACTOME 3 UTR MEDIATED TRANSLATIONAL REGULATION	**4.80E-04**	**-2.22**	**1.26E-04**	**-3.14**
REACTOME CHEMOKINE RECEPTORS BIND CHEMOKINES	**5.76E-04**	**-2.33**	**1.26E-04**	**-2.51**
KEGG RIBOSOME	**4.80E-04**	**-2.72**	**1.26E-04**	**-3.24**
REACTOME PEPTIDE CHAIN ELONGATION	**4.80E-04**	**-2.75**	**1.26E-04**	**-3.36**

Supplementary table 5: Pathways jointly associated to PER3 co-expressed genes in D1 and D2. Pathways enriched in PER3 positively co-expressed genes are shadowed in green whereas pathways negatively co-expressed with PER3 are shadowed in green.

D1: WGCNA analysis

A)

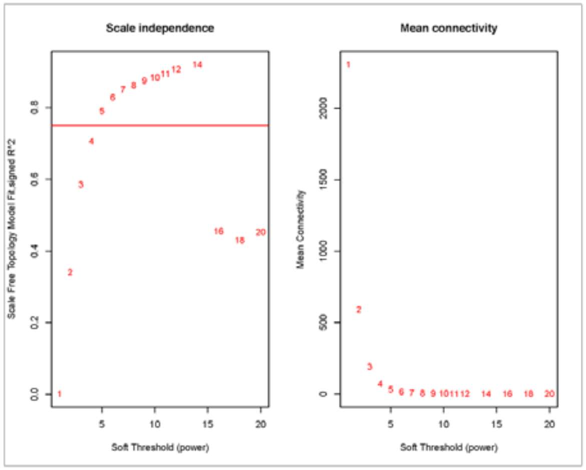

B)

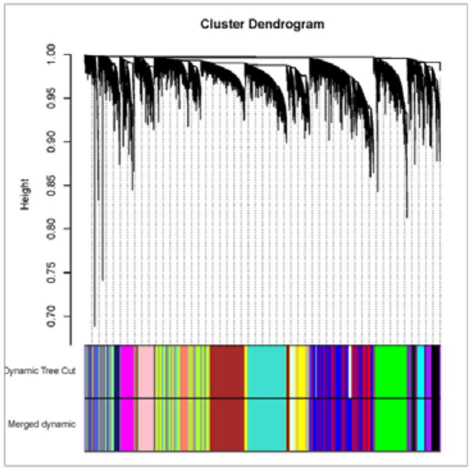

C)

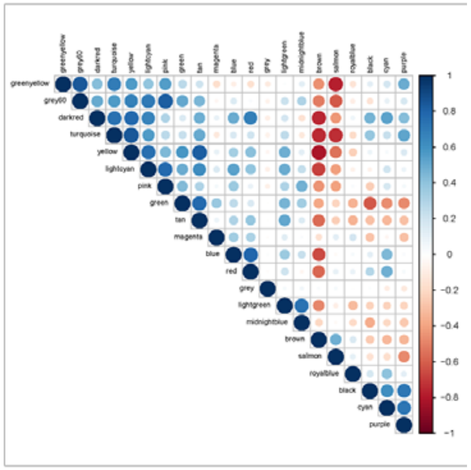

D2: WGCNA analysis

D)

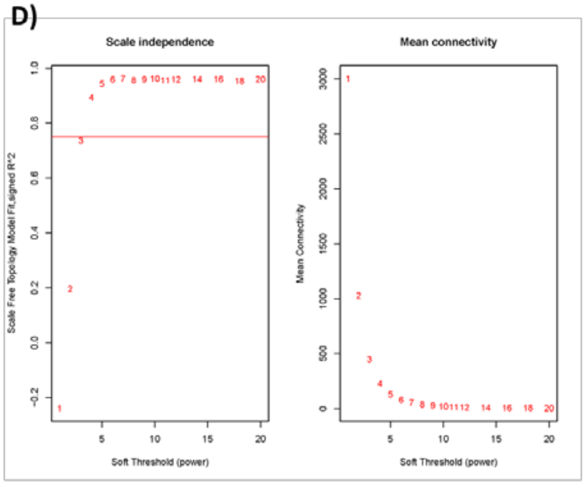

F)

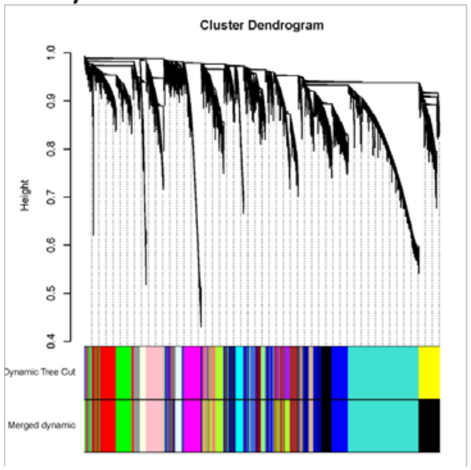

G)

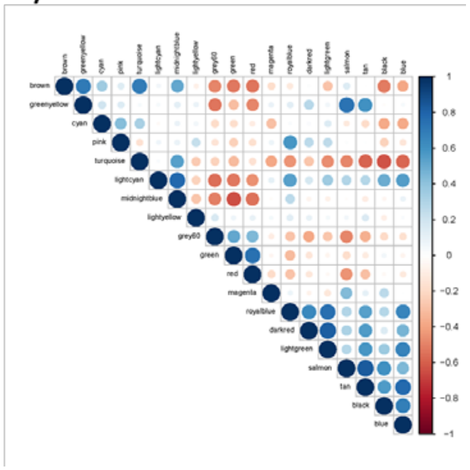

Supplementary figure 3: D1 and D2 WGCNA analysis results plots. A) D1 power selection. The leftmost plot shows the values of the power law fit depending on the power tested whereas the rightmost plot shows the mean connectivity based on the power selected. B) Modules detected by WGCNA method before and after the module merging procedure for the D1 healthy human mammary tissue

dataset. C) Module eigengen correlations in D1 healthy human mammary tissue dataset. D) D2 power selection. The leftmost plot shows the values of the power law fit depending on the power tested whereas the rightmost plot shows the mean connectivity based on the power selected. E) Modules detected by WGCNA method before and after the module merging procedure for the D2 healthy mice mammary tissue dataset. F) Module eigengen correlations in D2 healthy mice mammary tissue dataset.

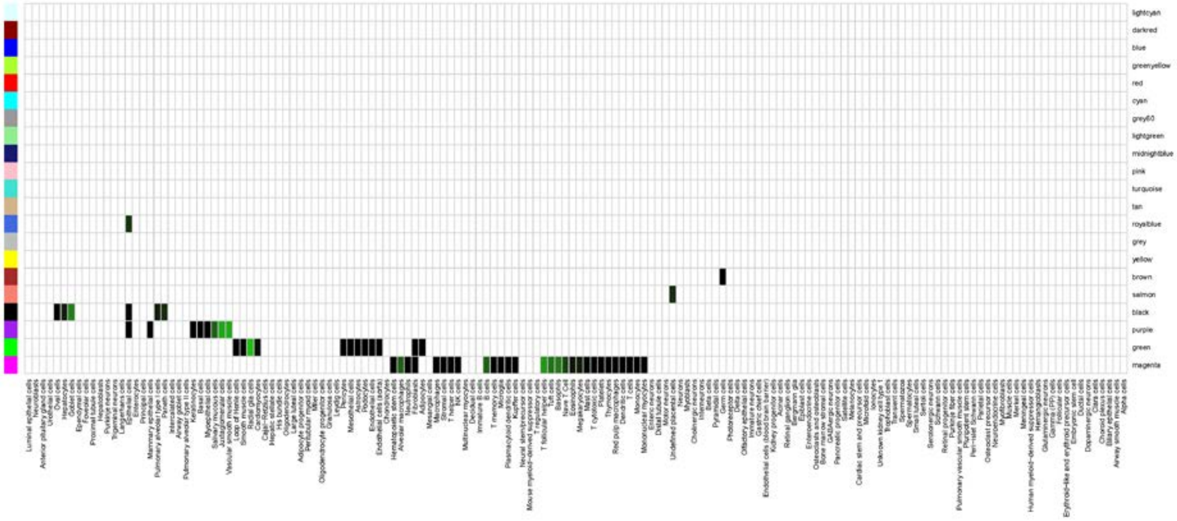

Supplementary figure 4: Enrichment in PanglaoDB cell type specific markers in the D1 human healthy mammary tissue dataset. Modules significantly enriched in specific cell type markers (p-adj < 0.05) are colored using a color range that goes from higher (green) lo lower (black) adjusted p-values.

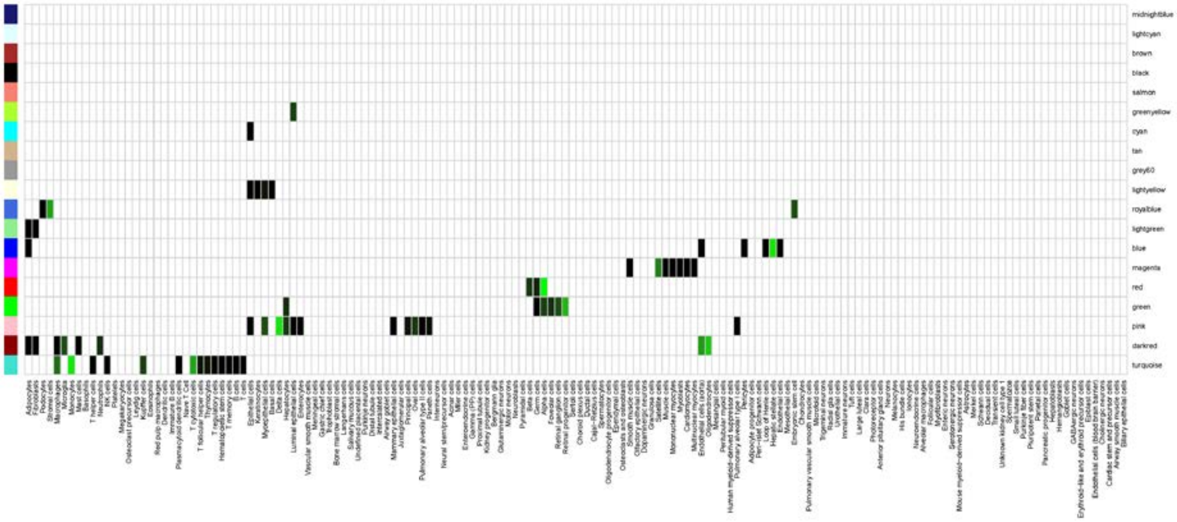

Supplementary figure 5: Enrichment in PanglaoDB cell type specific markers in the D2 murine healthy mammary tissue dataset. Modules significantly enriched in specific cell type markers (p-adj < 0.05) are colored using a color range that goes from higher (green) lo lower (black) adjusted p-values.

**Complete breast cancer dataset**		**Luminal A samples**	
**PER3**	221045_s_at 1	PER3	221045_s_at 1
**TEF**	225840_at	TEF	225840_at
**NR1D2**	225768_at	NR1D2	225768_at
**BNIP3L**	221478_at	BNIP3L	221478_at
**DBP**	201413_at	ASPH	225008_at
**AFF1**	201924_at	ARHGAP21	224764_at
**ASPH**	225008_at	HLF	204754_at
**ARHGAP21**	224764_at	FZD4	218665_at
**HLF**	204754_at	SSPN	226932_at
**FZD4**	218665_at	CRY2	212695_at
**PRKAR2B**	203680_at	IDH1	201193_at
**KCTD6**	238077_at	BCL2L2	209311_at
**SERINC1**	208671_at	MAOA	212741_at
**SSPN**	226932_at	SORBS1	218087_s_at
**PDE8A**	212522_at	TSPAN3	230282_at
**CRY2**	212695_at	HSDL2	209513_s_at
**IDH1**	201193_at	NFIA	226806_s_at
**BCL2L2**	209311_at	CAV2	203323_at
**TIMP3**	201150_s_at	PPARG	208510_s_at
**MAOA**	212741_at	SCP2	203445_s_at
**SORBS1**	218087_s_at	METTL7A	207761_s_at
**TSPAN3**	230282_at	KIAA0355	203288_at
**NFIA**	226806_s_at	FRY	204072_s_at
**PPARG**	208510_s_at	PTPRM	1555579_s_at
**SCP2**	203445_s_at	SAMD8	225950_at
**METTL7A**	207761_s_at	CDKN1C	213348_at
**PCOLCE2**	219295_s_at	SAMD10	227773_at
**KIAA0355**	203288_at		
**FRY**	204072_s_at		
**PTPRM**	1555579_s_at		
**SAMD8**	225950_at		
**CDKN1C**	213348_at		
**SAMD10**	227773_at		

Supplementary table 6: Genes and probes used to construct the average gene expression profiles for relapse-free survival analysis in the complete breast cancer dataset and the luminal A breast cancer analysis.
