# Supplementary material for: Circadian PERformance in breast cancer: a germline and somatic genetic study of PER3VNTR polymorphisms and gene co-expression
Source: NPJ Breast Cancer. 2021 Sep 10;7:118. doi: 10.1038/s41523-021-00329-2 (PMC8433453; doi:10.1038/s41523-021-00329-2)
Supplement: Supplementary file 2 — Reporting Summary [file 41523_2021_329_MOESM2_ESM.pdf]

nature portfolio

Corresponding author(s): Joan Climent Bataller

Last updated by author(s): Jul 30, 2021

Reporting Summary

Nature Portfolio wishes to improve the reproducibility of the work that we publish. This form provides structure for consistency and transparency in reporting. For further information on Nature Portfolio policies, see our Editorial Policies and the Editorial Policy Checklist.

Statistics

For all statistical analyses, confirm that the following items are present in the figure legend, table legend, main text, or Methods section.

n/a	Confirmed	
☐	☒	The exact sample size (*n*) for each experimental group/condition, given as a discrete number and unit of measurement
☒	☐	A statement on whether measurements were taken from distinct samples or whether the same sample was measured repeatedly
☐	☒	The statistical test(s) used AND whether they are one- or two-sided<br>*Only common tests should be described solely by name; describe more complex techniques in the Methods section.*
☐	☒	A description of all covariates tested
☐	☒	A description of any assumptions or corrections, such as tests of normality and adjustment for multiple comparisons
☐	☒	A full description of the statistical parameters including central tendency (e.g. means) or other basic estimates (e.g. regression coefficient) AND variation (e.g. standard deviation) or associated estimates of uncertainty (e.g. confidence intervals)
☐	☒	For null hypothesis testing, the test statistic (e.g. *F*, *t*, *r*) with confidence intervals, effect sizes, degrees of freedom and *P* value noted<br>*Give P values as exact values whenever suitable.*
☒	☐	For Bayesian analysis, information on the choice of priors and Markov chain Monte Carlo settings
☒	☐	For hierarchical and complex designs, identification of the appropriate level for tests and full reporting of outcomes
☐	☒	Estimates of effect sizes (e.g. Cohen's *d*, Pearson's *r*), indicating how they were calculated

Our web collection on statistics for biologists contains articles on many of the points above.

Software and code

Policy information about availability of computer code

Data collection	All analyses were carried out using R version 4.1
Data analysis	All analyses were carried out using R version 4.1

For manuscripts utilizing custom algorithms or software that are central to the research but not yet described in published literature, software must be made available to editors and reviewers. We strongly encourage code deposition in a community repository (e.g. GitHub). See the Nature Portfolio guidelines for submitting code & software for further information.

Data

Policy information about availability of data

All manuscripts must include a data availability statement. This statement should provide the following information, where applicable:

- Accession codes, unique identifiers, or web links for publicly available datasets
- A description of any restrictions on data availability
- For clinical datasets or third party data, please ensure that the statement adheres to our policy

All data generated during this study are included in this published article (and its supplementary information files). The data analyzed and not included in this paper are available on Gene Expression Omnibus repository (GEO, at https://www.ncbi.nlm.nih.gov/geo/). The GEO identifiers for the datasets analyzed are available on Supplementary Table 2 in the Supplementary File 1.

Field-specific reporting

Please select the one below that is the best fit for your research. If you are not sure, read the appropriate sections before making your selection.

☒ Life sciences ☐ Behavioural & social sciences ☐ Ecological, evolutionary & environmental sciences

For a reference copy of the document with all sections, see nature.com/documents/nr-reporting-summary-flat.pdf

Life sciences study design

All studies must disclose on these points even when the disclosure is negative.

Sample size	No sample-size calculation was performed. We used as many samples as we could gather together. In fact, to our knowledge this is the largest individual PER3VNTR and breast cancer association study to the date
Data exclusions	Samples with confusing genotype due to bad signal or bad DNA quality were excluded from the study.
Replication	A subgroup of samples was used to perform PER3VNTR genotyping by two different methods to double check and validate genotyping results. PCR products were resolved and separated in a 1.6% Electrophoretic gel and they were also runned on the “ABI PRISM 3730 DNA analyzer” and the results were analyzed using the GeneMapper Software (Life Technologies)
Randomization	NA
Blinding	PER3VNTR genotyping was blinded analyzed by three different researchers.

Reporting for specific materials, systems and methods

We require information from authors about some types of materials, experimental systems and methods used in many studies. Here, indicate whether each material, system or method listed is relevant to your study. If you are not sure if a list item applies to your research, read the appropriate section before selecting a response.

Materials & experimental systems

n/a	Involved in the study	
☒	☐	Antibodies
☐	☒	Eukaryotic cell lines
☒	☐	Palaeontology and archaeology
☒	☐	Animals and other organisms
☒	☐	Human research participants
☒	☐	Clinical data
☒	☐	Dual use research of concern

Methods

n/a	Involved in the study	
☒	☐	ChIP-seq
☒	☐	Flow cytometry
☒	☐	MRI-based neuroimaging

Eukaryotic cell lines

Policy information about cell lines

Cell line source(s)	We used DNA from the breast cancer cell lines to study the genotypes, however, we never used cell lines in culture. These DNA's were obtained at Allan Balmain's lab from the University of California San Francisco (UCSF).
Authentication	Cell lines were authenticated in the laboratory of origin at UCSF. We used DNA from those cell lines to genotype PER3VNTR. We did not run the autentification test in the DNAs since we never used cell lines in culture.
Mycoplasma contamination	We did not grow cell lines in culture
Commonly misidentified lines (See ICLAC register)	NA
